# Thermal Conductivity and Phase-Change Properties of Boron Nitride–Lead Oxide Nanoparticle-Doped Polymer Nanocomposites

**DOI:** 10.3390/polym15102326

**Published:** 2023-05-16

**Authors:** Bülend Ortaç, Saliha Mutlu, Taylan Baskan, Sevil Savaskan Yilmaz, Ahmet Hakan Yilmaz, Burcu Erol

**Affiliations:** 1 UNAM-National Nanotechnology Research Center and Institute of Materials Science and Nanotechnology, Bilkent University, Ankara 06800, Turkey; 2 Department of Chemistry, Faculty of Sciences, Karadeniz Technical University, Trabzon 61080, Turkey; 3 Department of Physics, Faculty of Sciences, Karadeniz Technical University, Trabzon 61080, Turkey; 4 Department of Physics, Faculty of Arts and Sciences, Recep Tayyip Erdoğan University, Rize 53100, Turkey

**Keywords:** thermal conductivity, phase-change materials, boron nitride–lead oxide polymer nanocomposite, polystyrene–polyethyleneglycol block copolymer, nanocomposite

## Abstract

Thermally conductive phase-change materials (PCMs) were produced using the crosslinked Poly (Styrene-block-Ethylene Glycol Di Methyl Methacrylate) (PS-PEG DM) copolymer by employing boron nitride (BN)/lead oxide (PbO) nanoparticles. Differential Scanning Calorimetry (DSC) and Thermogravimetric Analysis (TGA) methods were used to research the phase transition temperatures, the phase-change enthalpies (melting enthalpy (ΔH_m_), and crystallization enthalpies (ΔH_c_)). The thermal conductivities (λ) of the PS-PEG/BN/PbO PCM nanocomposites were investigated. The λ value of PS-PEG/BN/PbO PCM nanocomposite containing BN 13 wt%, PbO 60.90 wt%, and PS-PEG 26.10 wt% was determined to be 18.874 W/(mK). The crystallization fraction (F_c_) values of PS-PEG (1000), PS-PEG (1500), and PS-PEG (10,000) copolymers were 0.032, 0.034, and 0.063, respectively. XRD results of the PCM nanocomposites showed that the sharp diffraction peaks at 17.00 and 25.28 °C of the PS-PEG copolymer belonged to the PEG part. Since the PS-PEG/PbO and the PS-PEG/PbO/BN nanocomposites show remarkable thermal conductivity performance, they can be used as conductive polymer nanocomposites for effective heat dissipation in heat exchangers, power electronics, electric motors, generators, communication, and lighting equipment. At the same time, according to our results, PCM nanocomposites can be considered as heat storage materials in energy storage systems.

## 1. Introduction

Water and phase-change materials have been extensively studied in the literature as potential thermal energy storage media in construction applications. Water-based and PCM-based glass systems have been found to have much greater temperature-damping qualities than standard air-based glass systems [1]. By using the right cavity thickness, the storage system can be tailored for a certain climate zone [2]. Experimental studies found temperature damping to be promising in water-based systems [3]. Thermal energy storage (TES) is critical for the conservation of fossil fuels. New technologies, such as solar energy storage systems, are being introduced and studied in order to lower the energy demand of buildings [4]. In addition to batteries, Akr et al. investigated mechanical energy storage, thermal energy storage, magnetic energy storage, fuel cells, and energy storage technologies. A preliminary study and cost analysis, as well as appropriate building storage methods, will boost the efficiency of storage technology [5]. TES is a cutting-edge energy technology that is gaining traction in applications such as air and water heating, refrigeration, and air conditioning. TES appears to be the most appropriate mechanism for correcting imbalances between energy supply and demand [6]. Microencapsulated PCMs typically have a wall construction, whereas macroencapsulated PCMs can be embedded in floors and ceilings. Different researchers employ different approaches to studying the thermo-physical properties of new phase-change materials [7].

Phase-change Materials (PCMs) have a high capacity to store thermal energy. However, they have low thermal conductivity and poor heat transfer properties [8,9]. Therefore, heat transfer improvement techniques such as fins [10,11,12], metal foams [13,14,15], nano-additives [16,17,18] and encapsulation [19] are used to improve the heat transfer capabilities of PCMs. Khedher et al. investigated the effect of heat transfer on the thermal behavior of a closed environment filled with a neopentyl glycol/CuO solid–solid PCM nanocomposite and demonstrated that increasing the heat transfer rate using a fixed amount of material is an important task to improve fine performance [20]. In general, the addition of CNT nanoparticles, which have greater conductivity than Al_2_O_3_, to PCMs increases the effective thermal conductivity and surface area for heat conduction [21]. Meng et al. studied PCMs based on sodium sulfate decahydrate (Na_2_SO_4·_10H_2_O, SSD) as a thermal energy storage material. Alginate/SSD composite PCMs have been prepared by mixing SSD with different concentrations of alginate polymer [22]. Microcapsules have the ability to increase the thermal and mechanical performance of PCMs used in thermal energy storage, as they increase the heat transfer area and prevent leakage of melted materials [23]. Mohaddes et al. used a melamine formaldehyde (MF) resin as the shell material to encapsulate n-eicosane and showed that the latent heats of melting and crystallization of MF-based microcapsules were 166.6 J/g and 162.4 J/g, respectively [24]. They found that fabrics doped with such microcapsules exhibited a lower thermal lag efficiency and a higher thermoregulation capacity. PCMs are recognized as promising candidates for thermal energy storage that can improve energy efficiency in building systems. Li et al. designed and developed a new salt hydrate-based PCM composite with high energy storage capacity, relatively higher thermal conductivity, and excellent thermal cycling stability. The composite’s energy storage capacity and thermal conductivity are enhanced by the addition of various graphitic materials along with the Borax nucleator [18]. The use of PCMs provides higher heat storage capacity and more isothermal behavior during the charging and discharging state compared with sensible heat storage [25,26,27]. PCMs are chosen because of their use in various energy storage areas such as solar panels, waste heat recovery, and other heat energy storage systems [28]. Because the low thermal conductivity of PEG is undesirable in energy storage processes, many different studies have been carried out to overcome this disadvantageous situation [28,29,30]. These properties are fascinating for thermal interface materials [31,32]. Because PCMs are functional materials that can store and release large amounts of latent heat energy within a slight temperature change [33,34], they have been frequently used in solar energy storage [35], smart textiles [36,37,38,39], thermal protection of electronic devices [40], waste heat recovery [41] and smart housing [42,43,44].

This study used DSC and TGA to examine the thermal changes in the phase transitions of PS-PEG copolymers and PS-PEG/BN/PbO PCM nanocomposites. The ΔH_m_, ΔH_c_, T_m_, T_c_, and the decomposition temperatures of the PCM nanocomposites were investigated. ΔH_m_ and ΔH_c_ enthalpies of the PCM nanocomposites were investigated between −20–250 °C. The addition of PbO nanoparticles and BN nanoparticles to the copolymers increased the degradation temperature and residual amount of the polymers. For example, the PS-PEG (1000) polymer, which remained at 30.3 wt% at the 380 °C decomposition temperature, increased the decomposition temperature of the PCM nanocomposite to 402.9 °C, and that of the remaining composite amount to 51.820 wt%, as a result of 90% PbO nanoparticle addition. As a result of interactions between PbO nanoparticles and BN nanoparticles in PCM nanocomposites, F_c_ values were calculated to see how the crystallization rate changed [45,46,47]. The value of β in the equation is the mass fraction of the PS-PEG block copolymer [48]. Sun et al. [49] found that, as the ratio of additive materials in the PEG/CMPs composite increased, the F_c_ values were greater than the F_c_ value of PEG and were in the range of 102–105%.

Thermally conductive composite materials are produced using polymer materials with good machinability, low cost, and light weight [50,51,52]. Alizadeh et al. synthesized graft semi-interpenetrating polymer networks out of polyethylene glycol PEG 8000-based polyurethane and acrylic copolymers, and these graft-IPN samples can be used for thermal energy storage due to their high thermal properties [53]. Commercial grade polyethylene glycol (PEG) with a molecular weight of 6000 was tested by Sharma et al., and reliability tests of the PEG 6000 combined with techno-economic analysis have shown that this PCM can be used as a thermal energy storage system [54]. By creating nanodispersion polyethylene glycol (PEG)/PMMA/GnPs composites, Zhang et al. investigated the thermal and electrical properties of FSPCMs as well as their effects on morphology, structure, and form-stable performance [55]. The sol–gel coating method by Mo et al. obtained ternary lithium, sodium, and potassium carbonate/silica microcomposites as phase-change materials. It was concluded that microcomposites have an important place in high-temperature thermal energy storage [56]. Increased thermal conductivity of the PCMs was achieved by adding expanded graphite carbon nanotubes, graphene nanomaterials, activated carbon, carbon fiber, and metallic/oxide nanoparticles. BN is a universally accepted ceramic filler, especially for thermally conductive composites, due to its thermal conductivity and electrical insulator properties [57,58,59,60,61,62,63,64,65,66,67]. Qi and coworkers [61] increased the λ value of the PEG/graphene oxide (GO)/graphene nanoplatelet (GNP) PCM composite to 1.72 W/(mK) when they filled PEG using GO 2 wt% and GNP 4 wt% filler. Jia et al. added polyethylene glycol (PEG) to BN@CS scaffolds and showed that this increased the thermal conductivity value up to 2.77 W/(mK) [68]. While the thermal conductivity of pure PEG was measured at the level of 0.285 W/(mK), the thermal conductivity value for PEG@MXene was increased up to 2.052 W/(mK), with a 7.2-fold increase determined by Lu et al. [69]. The thermal conductivity of pure PCM and EHS/BNF composite PCMs containing 4 wt% were compared by Han et al., and the conductivity value of the EHS/BNF composite was determined to be 10.37 times higher than that of pure PCMs [26]. The thermal conductivities of pure PEG, PVA, PPVA, and the composite of GA/PEG as noted by Shen et al. [62] were 0.493, 0.152, 0.112 and 0.687 W/(mK). Polymers are flexible, light, durable, cheap, and resistant to abrasion and heat energy, and their usage has been increasing in every field from clothing to buildings and vehicles. In terms of developing the needed properties in structures, these characteristics highlight the important role of polymers in multidisciplinary scientific research [70,71,72]. The ability of a material to transfer heat energy is defined as thermal conductivity. We performed thermal conductivity calculations according to Equation (2) [73]. The thermal conductivity and the thermal properties of Portland Cement-HB (PAE-b-PCL)-PU plaster and HB (PAE-b-PCL)-PU/PbO-BN nanocomposites have been investigated by Cinan et al. [74]. When HB (PAE-b-PCL)-PU plaster, PbO, and AsO were added to Portland Cement, we found that they increased the properties of the cement, based on the thermal conductivity values. The thermal conductivity values of these PCMs were between 3.22 W/(mK) and 3.90 W/(mK) [72].

We have shown that PS-PEG copolymers doped with BN nanoparticles and PbO nanoparticles (i.e., PS-PEG PCM nanocomposites) are promising in terms of improving λ values and energy use efficiency. This article presents the preparation and thermal/physical characterizations of nano-enhanced PCMs with BN, a PbO nano blend, or single BN and PbO using crosslinked PS-PEG copolymers that we have previously synthesized and characterized [75,76,77]. The particle sizes of block copolymers and PCM nanocomposites were investigated using SEM and TEM analyses. At the same time, the XRD technique was used to determine the crystallographic structure of PCM nanocomposites.

Because of PCMs’ low thermal conductivity, their practical usage in latent heat storage units is limited. PS-PEG copolymers, PbO, and BN nanoparticles were utilized in this study, not only to boost thermal conductivity but also to develop PCMs with optimal compositions that can reduce latent heat. This study provides an outline of how phase-transition materials can be used in melting and solidification. The melting temperatures of the examined PCM nanocomposites ranged from 55.5 °C to 200 °C. As a result, the PCM nanocomposites can be used in high-temperature-operated absorption applications, cooling, waste energy production, and heat recovery operations. The results demonstrated strong intermolecular interactions between the PS-PEG copolymer, the BN, and the PbO nanoparticles and demonstrated that nanoparticle dispersion inside the PCM had no effect. The chemical structure of the nanoparticles was altered, but their thermal and chemical stability was improved. PCM nanocomposites were discovered to be more stable and to perform better thermally than PS-PEG copolymers. PS-PEGs have low thermal conductivity, which limits heat storage and release rates and limits their applicability. Greater thermal conductivity in PCMs reduces melting and solidification times and speeds heat transfer throughout these processes. The NCPSPB3 PCM nanocomposite has H_m_, T_m_, H_c_, and T_c_ values of 67.6 J g^−1^, 81.8 °C, 5.12 J g^−1^, and 188.4 °C, respectively. The ΔH_m_, T_m_, ΔH_c_ and T_c_ values of PEG/CNF [1], PEG/90CNF + 10rGONP [77], PEG1000 (45 wt%)/HNT-Ag^−1^ [1], PEG1000 (45 wt%)/HNT-Ag^−3^ [31] nanocomposites are 84.3 J g^−1^, 25.8 °C, 79.3 J g^−1^, 23.2 °C; 69.5 J g^−1^, 24.3 °C, 62.2 J g^−1^, 22.3 °C; 72.5 J g^−1^, 35.2 °C, 28.1 °C; 71.3 J g^−1^, 33.6 °C, and 25.7 °C, respectively. The enthalpy values of the NCPSPB3 PCM nanocomposite obtained in the study, PEG/CNF, PEG/90CNF + 10rGONP, the PEG1000 (45% wt%)/HNT-Ag^−1^ nanocomposites investigated by Zeighampour et al. [78], and the PEG1000 (45% wt%)/HNT-Ag^−3^ composite investigated by Song et al. [31] are near these values. PEG/CNF, PEG/90CNF + 10rGONP, PEG1000 (45 wt%)/HNT-Ag^−1^, and PEG1000 (45 wt%)/HNT-Ag^−3^ have thermal conductivity values of 0.68 Wm^−1^K^−1^, 0.85 Wm^−1^K^−1^, 0.73 Wm^−1^K^−1^, and 0.90 Wm^−1^K^−1^, respectively. The thermal conductivity value of the NCPSPB3 PCM nanocomposite is 27.30 times, 21.84 times, 25.43 times, and 20.63 times greater than the values of PEG/CNF, PEG/90CNF + 10rGONP, PEG1000 (45 wt%)/HNT-Ag^−1^, and PEG1000 (45 wt%)/HNT@Ag^−3^ composites, respectively. The H_m_, T_m_, H_c_, and T_c_ values of the NCPSPb8 and NCPSPbBN17 PCM nanocomposites are 43.8 J g^−1^, 83.8 °C, 4.94 J g^−1^, 185.5 °C, and 34.6 J g^−1^, 83.8 °C, and 5.36 J g^−1^, 190.2 °C, respectively. The values of PEG/50CNF + 50rGONP nanocomposite are 55.1 J g^−1^, 17.3 °C, 48.8 J g^−1^, 16.8 °C, which are comparable to those of the PEG/50CNF/50rGONP nanocomposite. The NCPSPb8 PCM nanocomposite, NCPSPbBN17 PCM nanocomposite, and PEG/50CNF + 50rGONP nanocomposite have thermal conductivity values of 17.14 Wm^−1^K^−1^, 15.71 Wm^−1^K^−1^, and 2.39 Wm^−1^K^−1^, respectively. PEG/50CNF + 50rGONP has a thermal conductivity value that is 7.17 times and 6.57 times lower than that of the NCPSPb8 and NCPSPbBN21 PCM nanocomposites, respectively. Song et al. [31] developed the PEG/HNT-Ag^−3^ nanocomposite PCM as a unique kind of stable nanocomposite PCM with a suitable phase-change temperature (33.6 °C), relatively significant latent heat (71.3 J g^−1^), outstanding thermal reliability, and increased thermal conductivity and conversion. Taking this into account, scientists demonstrated that it has a high potential for thermal energy storage and can be utilized as a building material to reduce indoor temperature changes, improve thermal comfort, and conserve electrical energy. According to Zeigampour et al. [78], SSPCNs with and without rGONP loadings have advantageous phase-transition temperatures, with latent heat values ranging from 55.1 to 84.3. They created SSPCNs that have shown excellent high-tech applications in accurate temperature control and quick temperature regulation [78]. The latent temperatures of various PCM nanocomposites ranged from 34.6 to 67.6 in this investigation. As a result, as described in the literature [31,78], these PCM composites can be used as building materials to reduce indoor temperature variations, increase indoor thermal comfort, conserve electrical energy, and provide precise temperature control and fast temperature regulation under certain conditions.

## 2. Materials and Methods

### 2.1. Materials

PbO Merck & Co. Inch. is produced and manufactured in Kenilworth, NJ, USA. BN particles of 1 µm size are an Aldrich product. PEG DM macrocrosslinkers were obtained from PEG polymers with molecular weights of 1000 gmol^−1^, 1500 gmol^−1^, and 10,000 gmol^−1^ by using methacrylic acid chloride [75,76,77].

### 2.2. Polymers

#### 2.2.1. Synthesis of the PEG DM Macrocrosslinkers and the PS-PEG Block Copolymers

The PEG DM macrocrosslinkers and the PS-PEG copolymers were synthesized according to [75,76,77].

#### 2.2.2. Preparation of the BN- and PbO-Doped PS-PEG PCM Nanocomposites

Table 1 shows the content of the PCM nanocomposites examined.

Known weights of PS-PEG block copolymers, PbO nanoparticles, and BN nanoparticles were mixed in an agar mortar and homogenized before being compressing into tablets. The tablets of the PCM nanocomposites were formed by hydraulic pressure at 10 MPa stress for 20 min at 22 °C. The tablet’s thickness was measured by using a BTS of 12,051 µm. The thickness of the tablets with a diameter of 12 mm ranges between 0.8–5 mm.

**Figure 1:**
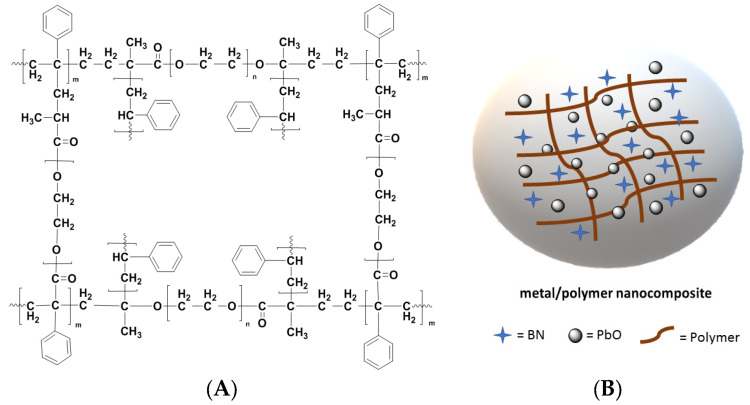
Schematic representation of the molecular formula (**A**) [74] of the PS-PEG block copolymer and (**B**) the PCM nanocomposite tablets.

Figure 1 shows the chemical structure of the polymers and the interaction between the polymers and nanoparticles.

### 2.3. Characterizations

The characterization of the macrocrosslinker synthesized according to the literature [67,68,69] was investigated by FT-IR, NMR, and GPC methods. PS-PEG block copolymers were investigated with FT-IR, SEM, and TGA instruments. The characteristic FT-IR peaks and properties of PEG DMs and PS-PEG block copolymers are similar to the results in the literature [67,68,69].

#### 2.3.1. Thermal Properties

##### TGA Method

The thermal decomposition process of the PEG-DM macrocrosslinkers, the PS-PEG block copolymers, and the PCM nanocomposites was implemented via the Seiko II Exstar 6000 TG/DTA (Seiko Instruments Inc., Chiba, Japan) analysis instrument. TGA thermograms of the macrocrosslinkers, block copolymers and PCM nanocomposites were obtained in a nitrogen gas atmosphere (200 mL/min) between 30–500 °C. The heating rate was taken as approximately 20 °C/min.

##### DSC Method

The T_m_, T_c_, ΔH_m_, and ΔH_c_ values of the PS-PEG copolymers and the PCM nanocomposites were obtained by using DSC (Perkin-Elmer Jade model, Perkin-Elmer Inc., Waltham, MA, USA). DSC measurements were made under nitrogen gas. The samples were examined at a heating rate of 10 degrees per minute from −20 °C to 300 °C and a cooling rate of 10 °C per minute from 300 °C to −20 °C. The weight of the PCM nanocomposites was approximately 3.0 mg. F_c_ values were calculated by using Equation (1) using DSC data [45,46,47].

(1) $$F_{c}=\frac{\Delta H_{\mathrm{PCMS}}}{\Delta H_{\mathrm{pure}}\beta }$$

Here, ΔH_pcms_ and ΔH_pure_ are the latent heat of the nanocomposite and the PS homopolymer, respectively. The value of ΔH_pure_ is 22.5 J/g. β is the mass fraction of the PS-PEG block copolymer in the nanocomposite.

### 2.4. Thermal Conductivity Method

By measuring the temperature difference between the two ends of the PCM nanocomposites, we calculated the thermal conductivity from Equation (2) [73]. We used a resistor that could go up to 50 W for 12 V to energize one surface of the sample. We determined the inlet temperature between 35−90 °C by applying 22.2 W of power.

(2) $$\lambda =\frac{q\left(x_{2}-x_{1}\right)}{A\left(T_{2}-T_{1}\right)}$$

Here, *λ* is the thermal conductivity, and its unit is given as W/(mK). The parameter q is the power of the resistor, given in Watts. *x*_1_ and *x*_2_ are the distance between the beginning and ends of the sample exposed to heat and the end of the heat flow, respectively. Equation (2), 𝑞 = −𝜆. 𝐴. ∇𝑇, is based on Fourier’s law. *T*_1_ > *T*_2_, since *T*_1_ is the initial temperature value applied to the *x*_1_ point of the sample. *T*_2_ is the temperature measured on the other end of the sample after the heat has passed through the sample. Therefore, *T*_2_ − *T*_1_ < 0.

### 2.5. Morphology

#### 2.5.1. SEM Analysis

The surface properties of the polymers and the PCM nanocomposites were elucidated with the SEM method. The SEM photographs were pictured by the JEOL JXA-840 brand model SEM instrument (Tokyo, Japan). The PS-PEG copolymers and the PCM nanocomposites were frozen with liquid nitrogen and then broken with an Edwards S 150 B model spray-coater. Broken specimens were plated with gold (300 Angstroms). SEM photos were taken at 10 kV, high vacuum, ESEM at 30 kV, and 3.0 nm resolution. The standard detectors used were ETD, low vacuum SED (LVD), gas SED for ESEM mode (GSED), and IR camera. Electron images from the cathode ray tube were recorded on a Polaroid film.

#### 2.5.2. TEM Analysis

The structural analyses of PbO and BN nanoparticles and the PCM nanocomposites were investigated by using the FEI-Tecnai G2F30 model TEM tool. The samples of which TEM pictures were taken were examined by fixing on carbon-coated TEM grids. The nanoparticles and PCM nanocomposites were imaged at 300 kV. The histogram of PbO nanoparticle sizes was obtained by counting more than 450 particles from the TEM image and using Image J Processing and Analysis software.

#### 2.5.3. XRD Method

The XRD data of the PCM nanocomposites were obtained with an X-Ray Diffractometer with trademark DMAX 2400 (Rigaku, Japan). The measurements were made under Copper K_α_ radiation with properties of 1.541 Å, 40 kV, and 100 mA. The measurements were taken at a scan rate of 8/min over 2 h and between 5–90 °C. The 2θ value and Miller indices of the PS-PEG block copolymers and the nanocomposite PCMs were investigated.

## 3. Results and Discussion

### 3.1. TGA Measurements of the PS-PEG-PbO and -BN Nanocomposite PCMs

**Figure 2:**
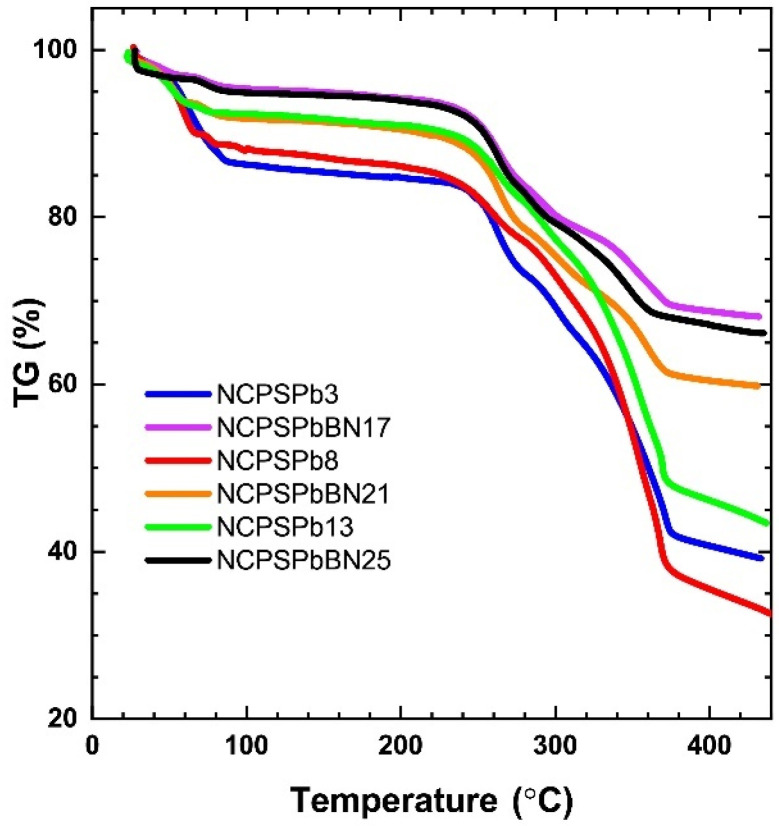
TGA thermograms of the NCPSPb3, NCPSPb8, NCPSPb13, NCPSPbBN17, NCPSPbBN21, and NCPSPbBN25 PCM nanocomposites.

Table 2 shows the degradation temperatures and the remaining mass (wt%) of the PCM nanocomposites. Figure 2 and Figure S1 show thermograms of PS-PEG (1000) PCMs with PbO additives. Figure 2 presents TGA thermograms of NCPSPb3, NCPSPb8, NCPSPb13, NCPSPbBN17, NCPSPbBN21, and NCPSPbBN25. Thermograms of other PCM nanocomposites are presented in Figures S1–S6. The thermograms demonstrate thermal degradations of all PCMs which were investigated between 40.3–402.9 °C. As a result, the thermal stability of the PCM nanocomposite with the addition of PbO nanoparticles is higher than the value of the polymers. This situation is due to increased physical interactions between PbO nanoparticles and PS-PEG polymer, such as van der Waals force and hydrophobic–hydrophobic interactions. The initial degradation temperature of NCPSPb3 PCM nanocomposite is higher than the value of NCPS1 and NCPSPb2. When the PbO content of the NCPSPb3 is increased and the polymer content is decreased, the moisture evaporates and the temperature decreases to 49.0 °C. NCPSPb3 has more PbO nanoparticles than the polymer, so its degradation temperature increases. The remaining masses of NCPSPb3 at its first and second degradation temperature are 96,900 wt% and 83,100 wt%, respectively. As a result, when PbO nanoparticles are doped into the PS-PEG (1000) copolymer (NCPS1), this increases the thermal stability of the polymer. As seen in Figure 2, Figures S1C, S2B, S3C, S4B, S5B and S6B, when NCPSPb4 contains PbO nanoparticles 70 wt% and 15 wt% BN, the amount of remaining mass is higher. TGA graphs of NCPSPb4 and NCPSPb5 are shown in Figure S1D and Figure S1E, respectively. The results of the NCPSPb5 are very different from the results of the NCPSPb2. Although the initial decay temperature is high in NCPSPb5, the remaining mass amounts are lower than in NCPSPb2. As a result, 53.8 wt% PbO nanoparticles in the NCPSPb5 decreased the thermal stability of the PCM nanocomposite.

The thermogram of the NCPS6 PCM nanocomposite is shown in Figure S2A. For NCPS6, the thermal stability, initial degradation temperature, and the amount of mass remaining after decomposition decreases as the molecular weight of the crosslinker increases, while the final degradation temperature increases. NCPSPb7 NCPSPb8, NCPSPb9 and NCPSPb10 graphs are presented in Figure S2B–E. When the PbO ratio is 50 wt% in the NCPSPb7, the initial degradation temperature is 50.7 °C, which is higher than the value of the NCPSPb2. As a result, thermal stability increased when the molecular weight of the crosslinker PEG was increased from 1000 to 1500, when the amount of PbO was the same. The initial decomposition temperatures and the amount of evaporated water of NCPSPb8 and NCPSPb9 containing 70 wt% and 90 wt% of PbO nanoparticles were decreased compared with the values of the NCPS6. As a result, the addition of a large amount of PbO for the PS-PEG-1500 polymer reduced thermal stability. The results for the NCPSPb10 are in agreement with the other nanocomposites.

Thermograms of NCPS11, NCPSPb12, NCPSPb13, NCPSPb14, and NCPSPb15 are shown in Figure S3A–E. Considering the degradation temperatures and the residual mass obtained for NCPSPb11-15 PCM nanocomposites, thermal stability decreased as the molecular weight of the macrocrosslinker increased from 1000 and 1500 to 10,000. However, an increased amount of PbO in PEG-10,000 indicates that it increased thermal stability.

The thermograms of NCPSBN16, NCPSPbBN17, NCPSPbBN18, and NCPSPbBN19 are presented in Figure S4A–D. When BN was added to the NCPS1 polymer in NCPSPb16, the thermal stability was observed to be higher than the thermal stability of NCPS1. As observed in NCPSPb17, thermal stability appears to be better than in NCPSPb16 when PbO is added. Increasing the PbO ratio to 90 wt% in the NCPSPb18 reduced the thermal stability of the composite to below the value of NCPSPb17.

Figure S1 and Figure 2 and show the thermal plot of PS-PEG (1500) doped with BN and PbO nanoparticles. The thermograms of NCPSBN20, NCPSBN21, NCPSBN22, and NCPSBN23 are shown in Figure S5A–D. When the TGA graphs of NCPSPb20, NCPSPb21 and NCPSPb22 nanocomposites are examined, it is clearly seen that increasing the amount of PbO nanoparticles in the PEG 1500 polymer increases its thermal stability. Considering the composition of the NCPSPb23 nanocomposite, its thermal stability is higher than that of NCPSPb21, because the amount of polymer is higher in its structure. However, its thermal stability is decreased a little due to the amount of PbO being lower than that in NCPSPb23.

The PCM nanocomposite thermograms (containing PS-PEG (10,000) block copolymer, and BN and PbO nanoparticles) are presented in Figure 2 and Figure S6. The graph for NCPSBN24 is shown in Figure S6A. The thermograms of NCPSPbBN25, NCPSPbBN26, and NCPSPbBN27 are shown in Figure S6B–D. It was observed that the addition of BN and PbO at different rates to the NCPSP11 nanocomposite (for NCSPb25 and NCPSPb26) increased the thermal stability. When the BN nanoparticle and PbO nanoparticle is below 15 wt% and 70 wt% (for NCPSPb27), respectively, the first decomposition temperature of the PCM nanocomposite is decreased, but the amount of the remaining mass does not change significantly. The remaining mass at the final decomposition temperature ranges from 68–74% in other samples, while it is around 50% for NCPSPb27.

The TGA results were examined in detail to illustrate the degradation of PbO- and BN-doped PS-PEG PCMs. As seen in all figures, there was little degradation when the PCMs were heated to about 430 °C, indicating that the PCMs are thermally stable. Three degradation temperatures were observed: 40 °C, 250 °C and 380 °C. The initial degradation of PS-PEG (1000) PCMs started at about 40 °C, and the final degradation was observed at 380 °C. In addition, the nanostructured BN particle additive yielded three degradation temperatures at 40 °C, 230 °C, and 330 °C. The TGA curves of all the PbO–BN-doped nanocomposite PCMs exposed thermal degradation actions parallel to that of the PbO-doped PS-PEG (1000) PCMs. A few degradation points were between 50–380 °C. The initial loss began at 50 °C and the last degradation at 380 °C originated from the degradation of the PS-PEG (1500) PCMs. In addition, the nanostructured BN nanoparticle additive showed three degradation temperatures around 40–240 °C and 420 °C. The first stage of degradation started at 40 °C and the final decomposition temperature was 430 °C, which caused the degradation of the PS-PEG copolymer in PCMs. Furthermore, the addition of BN nanoparticles showed several degradation temperatures in the range of 40 °C to 420 °C. As a result, it was seen that losses between 40 °C and 80 °C in all composites were caused by the evaporation of water. In addition, PCMs with higher PbO and BN nanoparticle ratios represent thermal stability. This is due to the large number of nanoparticles in PCMs that prevent the degradation of polymer chains. The present results show that the PS-PEG polymer becomes more thermally stable when the PCM nanocomposites are incorporated with PbO and BN nanoparticles. Because of these uses, PCM nanocomposites can exhibit excellent thermal persistence for a variety of energy application systems.

### 3.2. DSC Results of the PS-PEG/BN/PbO PCM Nanocomposites

**Figure 3:**
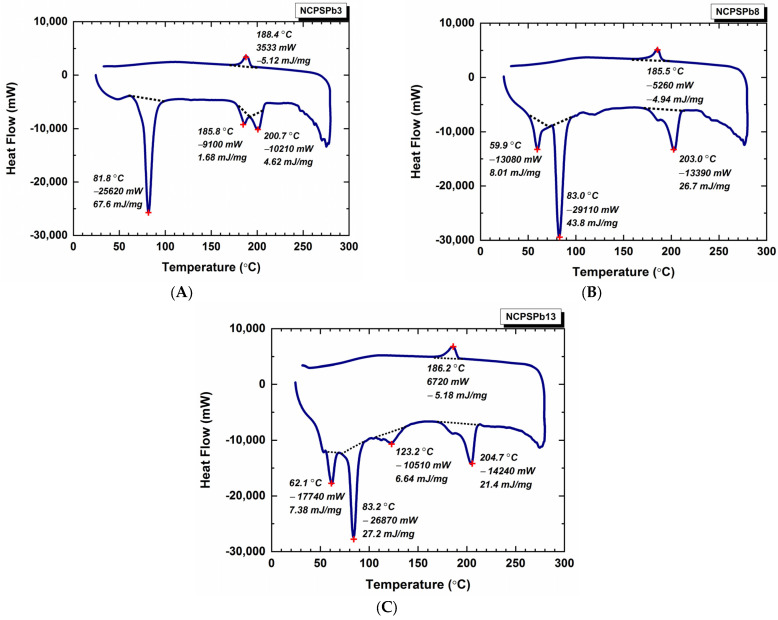
Thermal behavior of PS-PEG PCMs: (**A**) NCPSPb3, (**B**) NCPSPb8, (**C**) NCPSPb13.

**Figure 4:**
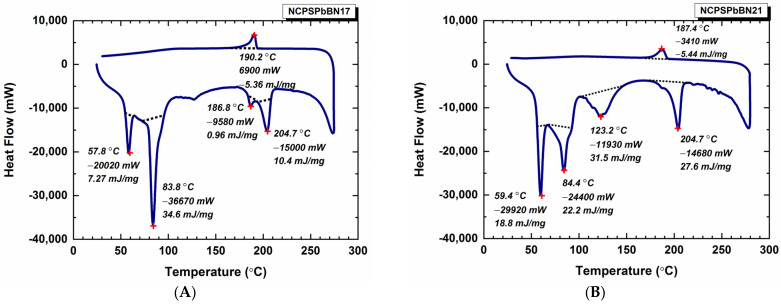

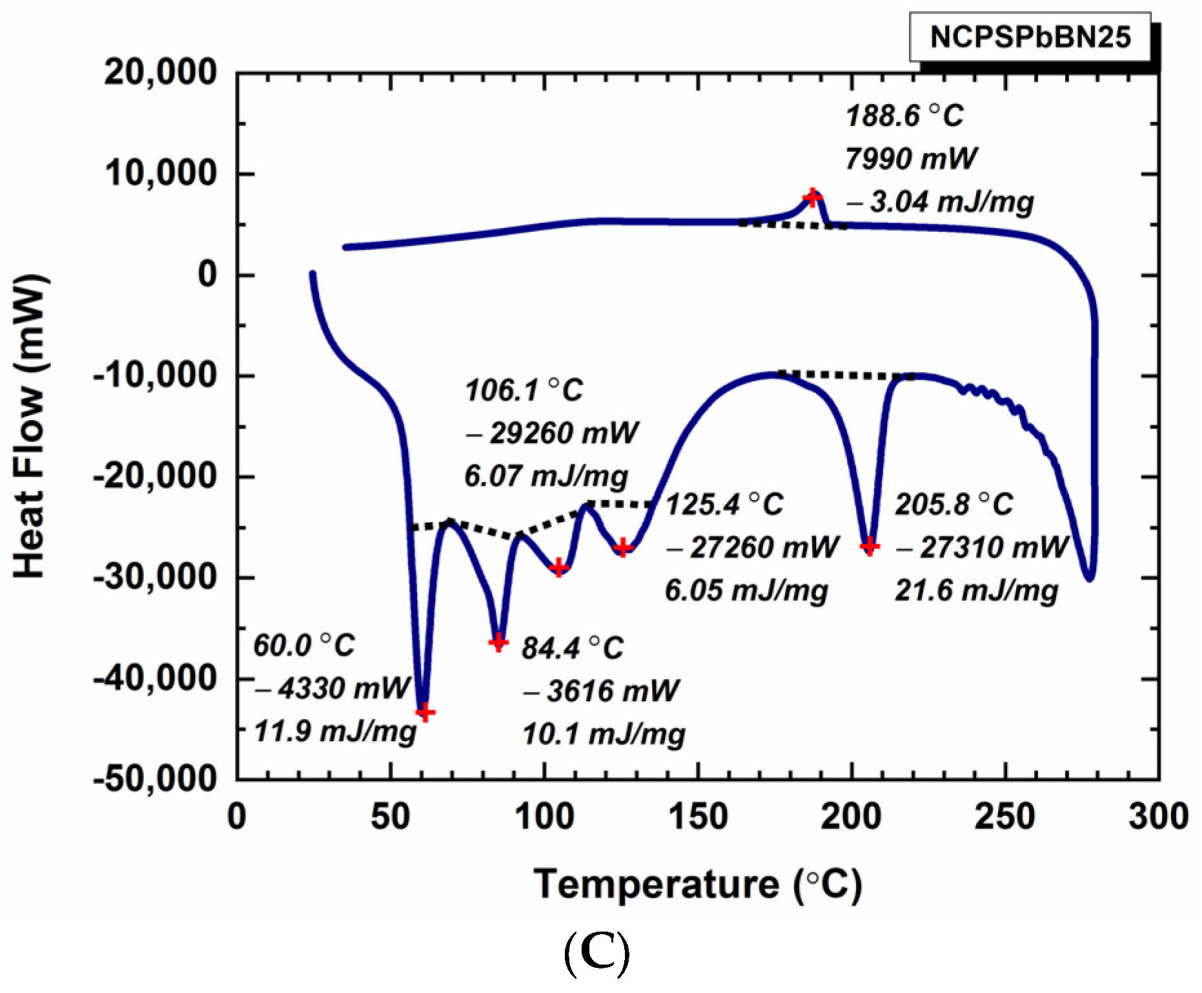
Thermal behavior of PS-PEG PCM nanocomposites: (**A**) NCPSPbBN17, (**B**) NCPSPbBN21, (**C**) NCPSPbBN25.

Phase-change temperatures of PS-PEG/PbO, PS-PE/BN, and PS-PEG/PbO/BN PCM nanocomposites were measured by the DSC technique. Melting and solidification DSC curves of PS-PEG PS-PEG/PbO, PS-PE/BN, and PS-PEG/PbO/BN PCM nanocomposites are shown in Figure 3 and Figure 4. The T_m_, ΔH_m_, T_c_, ΔH_c_ values of the PS-PEG PCM nanocomposites were determined from the endothermic and exothermic curves during phase change. The melting/solidification temperature (T_m_/T_c_), endothermic/exothermic enthalpy (ΔH_m_/ΔH_c_) and F_c_ values are given in Table 3. T_m_-T_c_, ΔH_m_-ΔH_c_ and F_c_ values are the most effective ways to interpret the thermal behavior of PCM nanocomposites. Endothermic peaks were observed between 55.5–205.8 °C due to melting (as seen in Table 3 and Figure 3 and Figure 4).

Figure 3 shows the thermal behavior of NCPSPb3, NCPSPb8, and NCPSPb13 PCM nanocomposites, while those of other PCM nanocomposites are given in Supplementary S2. Figure S7 presents the thermal behavior of NCPS1, NCPS6 and NCPS11. Compared with the NCPS1 (PS-PEG 1000) polymer, the T_c_ of the NCPSPb3 PCM nanocomposite slightly increased. A significant increase was observed in ΔH_m_ values (Figure 3A and Figure S2A). DSC curves of NCPSPb8 and NCPS6 are seen in Figure 3B and Figure S7B. As can be seen from these figures, the addition of 70% by weight of PbO to the NCPS6 block copolymer caused a partial increase in T_m_ values of NCPSPb8. It also greatly increased the ΔH_m_ values. T_m_ and ΔH_m_ values of NCPSPb13 and NCPS11 are shown in Figure 3C and Figure S7C. The average ΔH_m_ value corresponding to melting temperatures of the NCPS11 in the range of 58.8–190.1 °C was 10.3 J g^−1^. The ΔH_c_ value at 31.5 °C was found to be −7.98 J g^−1^. T_m_-ΔH_m_ values of NCPS13 were 62.1 °C–7.38 J g^−1^, 83.2 °C–27.2 J g^−1^, 123.2 °C–6.64 J g^−1^, and 204.7 °C–21.4 J g^−1^. T_c_-ΔH_c_ values of NCPS13 were 186.2 °C, −5.18 J g^−1^.

T_m_ values of NCPSBN16 (in Figure S8A) are shown in Table 3. When the NCPS1 results are compared with those of NCPSBN16, their T_m_ values were found to be not much different. As can be seen from the data in Table 3, the addition of BN nanoparticles to the PS-PEG polymer increases the ΔH_m_ values. T_m_-ΔH_m_ values of NCPSPbBN17 can be seen in Figure 4A. It is evident that the incorporation of the filler into the matrix increases the T_m_ of the PCM nanocomposites. T_m_ values of the NCPSBN20 (in Figure S8B) showed minor fluctuation changes compared with the NCPS6 block copolymer. DSC thermograms of the NCPSPbBN21, NCPSBN24, and NCPSPbBN25 PCMs are shown in Figure 4B, Figure S8C, and Figure 4C, respectively. The average ΔH_m_ value for the four T_m_ values corresponding to the 59.4–204.7 °C range of the NCPSPbBN21 is 25.0 J g^−1^, and the ΔH_c_ value corresponding to the T_c_ (187.4 °C) temperature is −5.44 J g^−1^. For NCPSBN24, the mean ΔH_m_ value corresponding to the temperatures of 55.5−184.8 °C was found to be 15.2 J g^−1^. The ΔH_c_ value at the T_m_ of 112.1 °C is 0.62 J g^−1^. For NCPSPbBN25, the mean ΔH_m_ value corresponding to five melting temperatures was found to be 11.1 J g^−1^ and the ΔH_c_ value was found to be 3.04 J g^−1^ at T_c_ = 188.6 °C. Adding a certain amount of BN nanoparticles to the PCM nanocomposite causes the composite to shift to lower melting and crystallization transition temperatures. T_m_ and T_c_ values of the NCPS1, NCPS6, and NCPS11 copolymers were found to increase with increasing molecular weight of the PEGs. It was observed that T_c_ and T_m_ values increased with the addition of PbO nanoparticles in NCPSPb3, NCPSPb8, and NCPSPb13. When BN particles are added to NCPS1, NCPS6, and NCPS11 block copolymers, T_c_ values decrease while T_m_ values are almost the same. The melting and cooling curves of the selected sample composites were formed in almost the same regions as the DSC curves in Figure 3, Figure 4, Figures S7 and S8. In this case, it can be concluded that PCM nanocomposites have a similar phase change.

ΔH_m_ is important for PCMs containing PEG [41]. ΔH_m_ values decreased for BN/PS-PEG nanocomposites. This decrease led to a decrease in ΔH_m_ as a result of steric effects that change the structure of the polymer chains and the increase in the mobility and free volume of the polymer matrix at temperatures above T_m_. These effects are due to the decrease in T_m_ of the PS-PEG/BN nanocomposite. In addition, the interactions between the nanoparticles and the polymer matrix reduced the free volume of the polymer chain [49,72]. When PbO and BN nanoparticles are homogeneously dispersed in the PS-PEG matrix, close interactions such as surface tension forces, π–π interactions and capillary forces between nanoparticles and PS-PEG will limit the mobility of the PS-PEG polymer. This causes a decrease in the phase-change temperature. The highest phase-change enthalpy of the PCM nanocomposite containing PbO 70 wt% and PS-PEG (1000) 30 wt% is 67.6 J g^−1^. In addition, the thermal conductivity of composites can be significantly increased by the addition of PbO and BN nanoparticles, which leads to a fast thermal response.

As a result, the T_m_, ΔH_m_, T_c_ and ΔH_c_ values of the PCM nanocomposites that we examined fall within the range of the PEO-CMC, PEO-CEL [79], AMPD/TAM, NPG/TAM/PE/AMPD [80], NPG/PE [81], and PE-TAM [82] composites.

In addition, F_c_ values were calculated for different compounds using the crystallization enthalpy values obtained from DSC (Table 3). It was observed that the F_c_ values calculated from the DSC result increased. The F_c_ value of the PS-PEG copolymer increases as the ratio of BN nanoparticles and PbO nanoparticles increases. The F_c_ value of NCPSPb8 increases to 0.724 with the addition of PbO nanoparticles into NCPS6, whose F_c_ value is 0.063. When BN nanoparticles are added to NCPS6, the F_c_ value of 0.063 increases to 0.094 and 0.092 (NCPSBN20), and when PbO is added to this composite, the ratio increases even more, with a value of 1.777 obtained for NCPSPbBN21. We observed the same effect in all our other samples as follows: F_c_ values are 0.032 and 0.034 for pure NCPS1. These values increased with the contribution of PbO and reached 0.728. Only with the BN additive does the F_c_ value rise to 0.089 and 0.087, and when pure PS-PEG is added to BN nanoparticles and PbO nanoparticles, the F_c_ value reaches 1.985. The F_c_ value of the NCPS11 copolymer is 0.305. With the addition of PbO nanoparticles to the NCPS11 copolymer, the F_c_ value increases to 0.698. At the same time, if the BN nanoparticle and PbO nanoparticle are used together, the F_c_ value reaches 0.860.

The measured latent heat capacity of the 70 wt% PbO-nanoparticle-doped NCPSPb3 composite is 89.5% larger than the value of NCPS1. The latent heat capacity of NCPSPb8 doped with 70 wt% PbO nanoparticles is 92% higher than that of its polymer. This possibility is attributed to the scarcity of physical interactions between nanoparticles and PS-PEG, such as van der Waals force and hydrophobic–hydrophobic interactions, which can restrict the mobility of PS-PEG molecular chains during the crystallization process. As a result, the phase-change enthalpy of NCPSPB3, NCPSPb8, and NCPSPb13 containing PS-PEG and PbO nanoparticles increases. As the PbO and BN nanoparticle content increases in PCM nanocomposites, the thermal conductivity increases. Also, the latent heat gradually increases. This implies that increasing thermal conductivity using PbO and BN nanoparticles will be accompanied by increased latent heat in the nanocomposites. In this study, PbO and BN nanoparticles led to an increase of 61.9% in thermal conductivity and 93.8% in latent heat of PS-PEG block copolymers. Therefore, it would be beneficial to add PbO and BN nanoparticles at a low loading rate to obtain the appropriate latent heat and to increase the thermal conductivity of the composites.

### 3.3. Thermal Conductivity

λ values were investigated for nanocomposites prepared from different amounts of PS-PEG copolymer, BN nanoparticles, and PbO nanoparticles. To determine the thermal conductivity of PCM nanocomposites, 5 wt%, 13 wt%, 15 wt%, and 50 wt% of BN nanoparticles and 10 wt%, 53.8 wt%, 70 wt%, 90 wt% of PbO nanoparticles were used.

**Figure 5:**
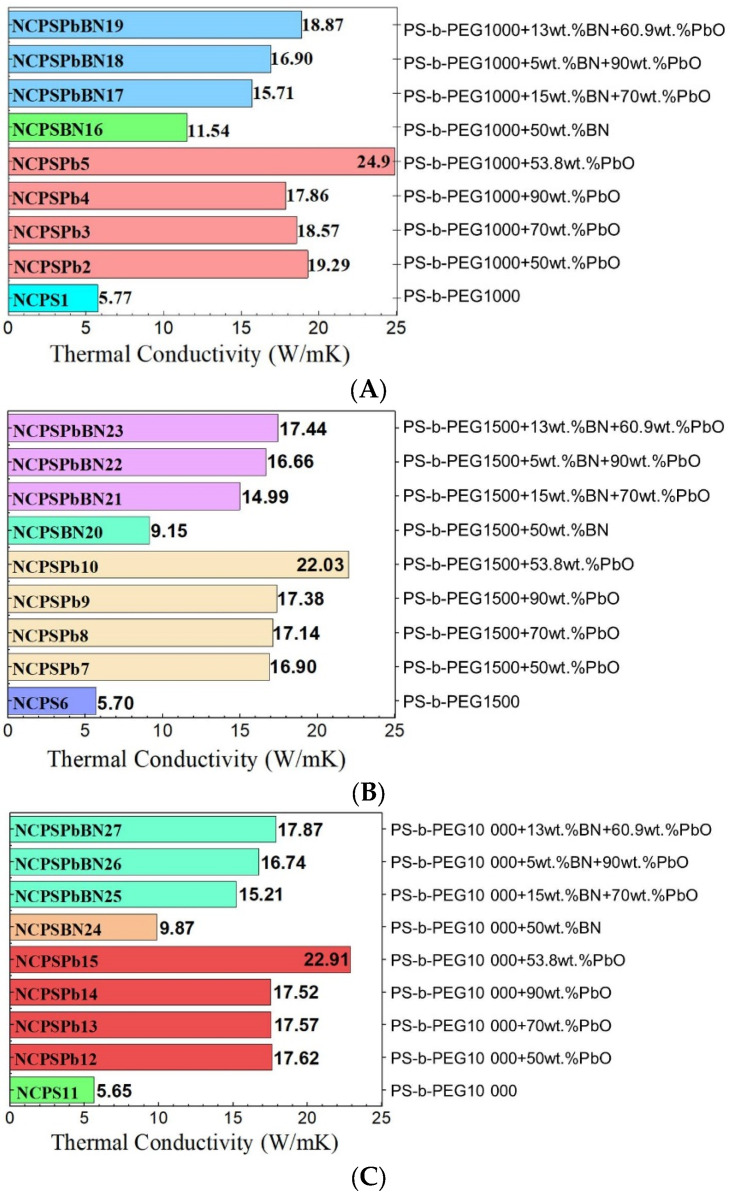
λ values of the (**A**) PS-PEG (1000) copolymers and PS-PEG (1000)/PbO and PS-PEG (1000)/PbO/BN PCM nanocomposites; (**B**) PS-PEG (1500) copolymers, PS-PEG (1500)/PbO and PS-PEG (1500)/BN PCM nanocomposites; and (**C**) PS-PEG (10,000) copolymers and PS-PEG (10,000)/PbO and PS-PEG (10,000)/PbO/BN PCM nanocomposites.

The results of the λ values according to the additive ratios are given in Figure 5A–C. The λ values of PS-PEG copolymers and the PCM nanocomposites were calculated according to Equation (2). When the λ values of PS-PEG copolymers and PCM nanocomposites are examined from Figure 5, it is seen that the λ values of PS-PEG/PbO PCM nanocomposites are higher than that of the PS-PEG block copolymer. The greater value of PCM nanocomposites compared with their copolymers is due to the increase in free volume with the addition of PbO nanoparticles. The λ value of the NCPS1 block copolymer (PS-PEG (1000) was found to be 5.77 W/(mK)). The λ values of NCPSPb2, NCPSPb3, NCPSPb4, and NCPSPb5 containing the PS-PEG (1000) copolymer were 234%, 222%, 210%, and 332% higher, respectively, than the λ value of the copolymer (Figure 5A). The λ values increased with the contribution of 50–90 weight PbO nanoparticles to the NCPS1 polymer. It was observed that the increase in λ value of the NCPSPb5 nanocomposite was higher than that of the polymer. The λ value of NCPS6 (PS-PEG (1500) block copolymer) was found to be 5.70 W/(mK). The λ values of NCPSPb7, NCPSPb8, NCPSPb9, and NCPSPb10 were 196%, 201%, and 286% higher than the λ value of its copolymer. Among the PbO nanoparticle-doped PS-PEG (1500) PCM nanocomposites, NCP-SPb10 (46.2 wt% PS-PEG (1500) and 53.8 wt% PbO nanoparticles) had the highest λ value. The λ value of NCPS11 (PS-PEG (10,000) block copolymer) was 5.65 W/(mK). The λ values of NCPSPb12, NCPSPb13, NCPSPb14, and NCPSPb15 (50 wt%, 70 wt%, 90 wt%, and 53.8 wt% of PbO nanoparticles) were 212%, 211%, 210% and 305% higher, respectively, than the λ value of NCPS11 (Figure 5C). The λ value of the NCPSPb15 nanocomposite (22.91 W/mK) was much higher than that of the NCPS11 block copolymer. The λ value of NCPSBN16 was calculated as 11.54 W/(mK). When 50 wt% BN nanoparticles were added to the thermal conductivity of the NCPS1 copolymer, the λ value increased from 5.77 W/(mK) to 11.54 W/(mK). The λ graphs of NCPSPbBN17, NCPSPbBN18, and NCPSPbBN19 are presented in Figure 5A, and the λ values of these PCM nanocomposites are n 15.71 W/(mK), 16.9 W/(mK) and 18.87 W/(mK), respectively. NCPSBN16, NCPSBN20, and NCPSBN24 contain the addition of 50 wt% BN to PS-PEG (1000), PS-PEG (1500), and PS-PEG (10,000) block copolymers. When the λ values of these composites were compared with the λ values of the copolymers, BN nanoparticle addition increased the thermal conductivity of the block copolymers by 100%, 61%, and 75%, respectively. It is the NCPSPbBN23 nanocomposite which has the highest thermal conductivity (17.44 W/(mK)) among NCPSPbBN21, NCPSPbBN22, and NCPSPbBN23. As a result of the incorporation of PbO and BN nanoparticles into NCPSPbBN21, NCPSPbBN22, and NCPSPbBN23 PCM nanocomposites, the λ values of the composites increased by 163%, 193%, and 206% from the λ values of their copolymers (Figure 5B). The λ values of the NCPSPbBN25, NCPSPbBN26, and NCPSPbBN27 PCM nanocomposites were 15.21 W/(mK), 16.74 W/(mK), 17.87 W/(mK). NCPSPbBN27 produced the highest λ value among NCPSPbBN25, and NCPSPbBN26. The λ values of NCPSPbBN25, NCPSPbBN26, and NCPSPbBN27 were increased by 169%, 196%, and 216%, respectively, from the thermal conductivity values of their copolymers when PbO and BN nanoparticles are added (Figure 5). As a result, the BN additive increased the thermal conductivity values of the nanocomposites. Composites with added PS-PEG and PbO produce the best value every time (with PS-PEG 46.2 wt% and PbO 53.8 wt%). The PCM nanocomposites prepared from synthesized PS-PEG (1000, 1500, 10,000) copolymers can be used for thermal conductivity. Using BN and PbO nanoparticles thermal conductive fillers, Zheng et al. produced a new type of energy storage material with high thermal conductivity by adding different masses of hydroxylated multi-walled carbon nanotubes (MWCNTs) to the stable form of the PEG1500·CaCl_2_ phase-change material [82]. We observed that the phase change and thermal conductivity values of the PS-PEG copolymers that we synthesized here were as high as in those studies. 

We also used BN nanoparticles and PbO nanoparticles to solve the low heat dissipation problem of PS-PEG polymer materials. We show that PS-PEG block copolymers are important in the thermal conductivity of PCM nanocomposites. Pure Pb metal has a high λ value and was found as 35 W/(mK) in the temperature range of 20 °C–85 °C [83]. As a result, since oxides are useful materials for thermal barriers and form rich structures, this allows them to increase the thermal conductivity of the high compositions and the high efficiency in forming composites. We obtained high conductivity values by using PbO nanoparticles in our study. The λ value for PbO nanoparticles was 17.5 W/(mK) [84]. Based on the work of Zhou et al., the λ value for BN was 2 W/(mK) [72].

Since the λ value of polymers can be increased by using various materials [84,85,86,87], it was concluded that the λ value of nanocomposites formed by adding PS-PEG copolymers and BN and PbO nanoparticles increased, Lebedev’s study presented that the thermal conductivity values of LLDPE and PLA-based composites increased 1.9 and 3.5 times with 40% filler [72,88]. The thermal conductivity of materials is also important for heat performance, which is a way to conserve energy to increase the efficiency of the systems. Phase-change energy storage technologies are developing. Polymers with phase-change properties are far from the desired performance due to their thermal conductivity. It is common to use additional fillers to improve the performance of polymers. Therefore, the composites we prepared have reached the desired level.

The λ value of the PS-PEG/BN PCM nanocomposite was found to be increased by 63% compared with the λ value of the PS-PEG copolymer. In addition, the λ value of the PS-PEG (1000)/PbO PCM containing PbO 53.8 wt% and PS-PEG 46.20 wt% is 24.90 W/(mK). The λ value of the PCM nanocomposite doped with PbO nanoparticles was found to be 332% higher than that of the PS-PEG (1000) block copolymer. The thermal conductivity of PS-PEG/PbO PCM nanocomposites containing PbO nanoparticles 50 wt%, 70 wt%, and 90 wt% were higher than the thermal conductivity values of their copolymers. It was found that the thermal conductivity values of PS-PEG (1000, 1500, 10,000)/PbO PCM nanocomposites (containing PbO nanoparticles 53.8 wt%) were increased by 332%, 291%, and 305% from their block copolymer, respectively. The results also showed that BN nanoparticles and PbO nanoparticles significantly increased the thermal conductivity of the PCM nanocomposites.

### 3.4. Morphology Results

#### 3.4.1. SEM Images of the PCM Nanocomposites

**Figure 6:**
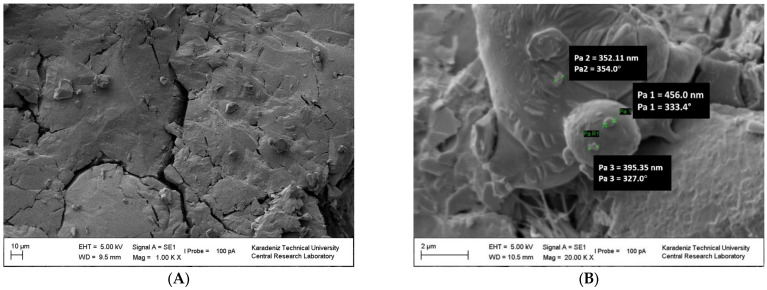
SEM images of (**A**) NCPS6 and at (**B**) NCPSPb3.

**Figure 7:**
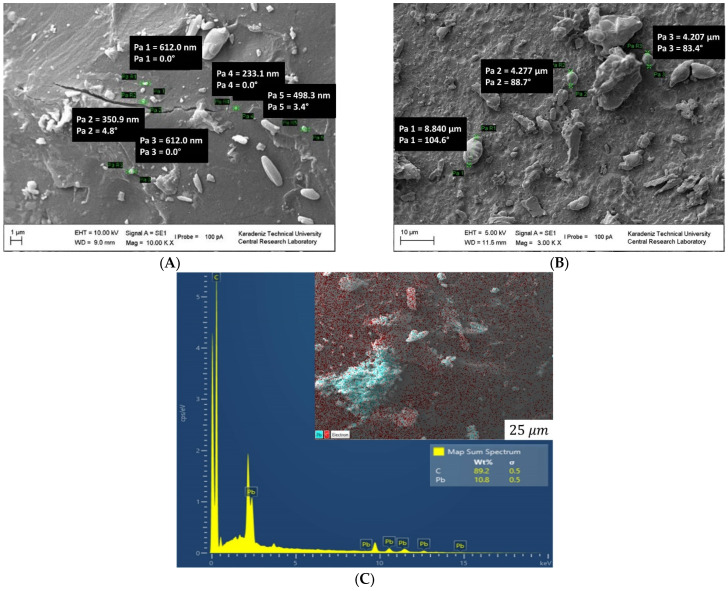
SEM images of NCPSPb8 (**A**) and NCPSPb13 (**B**). EDS mapping and analysis indicating the element distribution on the surface of the NCPSPb8 nanocomposite (**C**).

**Figure 8:**
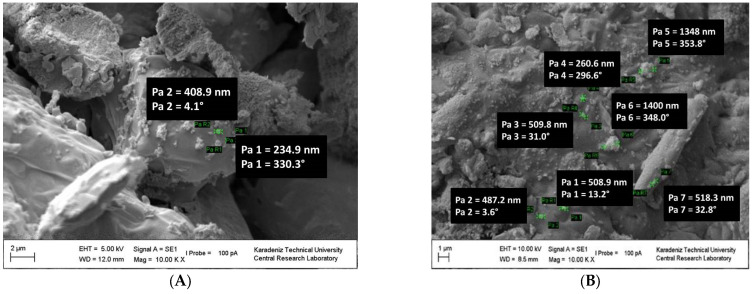

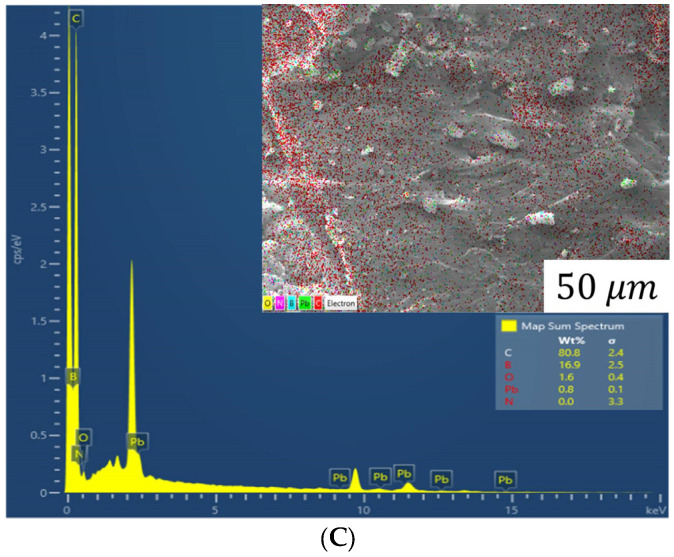
SEM images of NCPSPbBN17 (**A**) and NCPSPbBN21 (**B**). EDS mapping and analysis indicating the element distribution on the surface of the PS-PEG-BN-PbO nanocomposite and SEM images of NCPSPbBN21 at (**C**).

**Figure 9:**
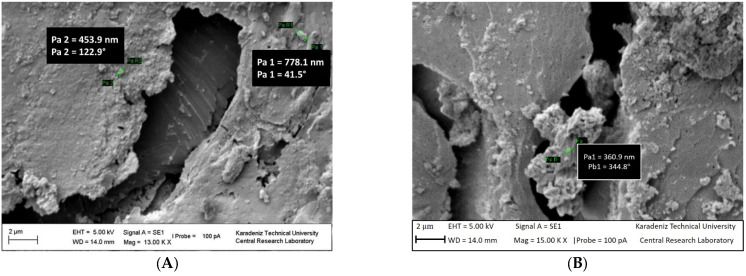
SEM images (**A**,**B**) of the NCPSPbBN25 nanocomposite.

**Figure 10:**
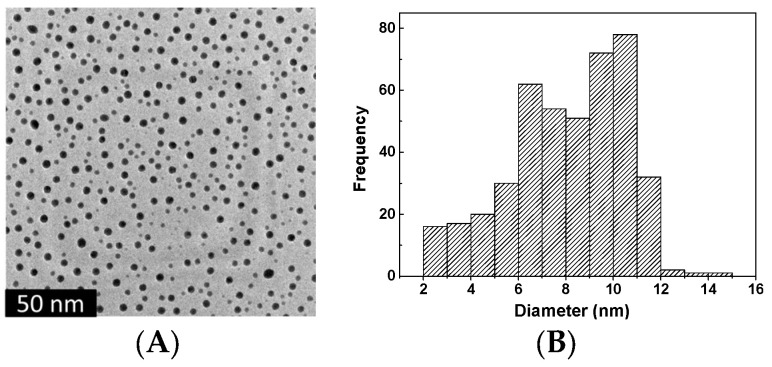

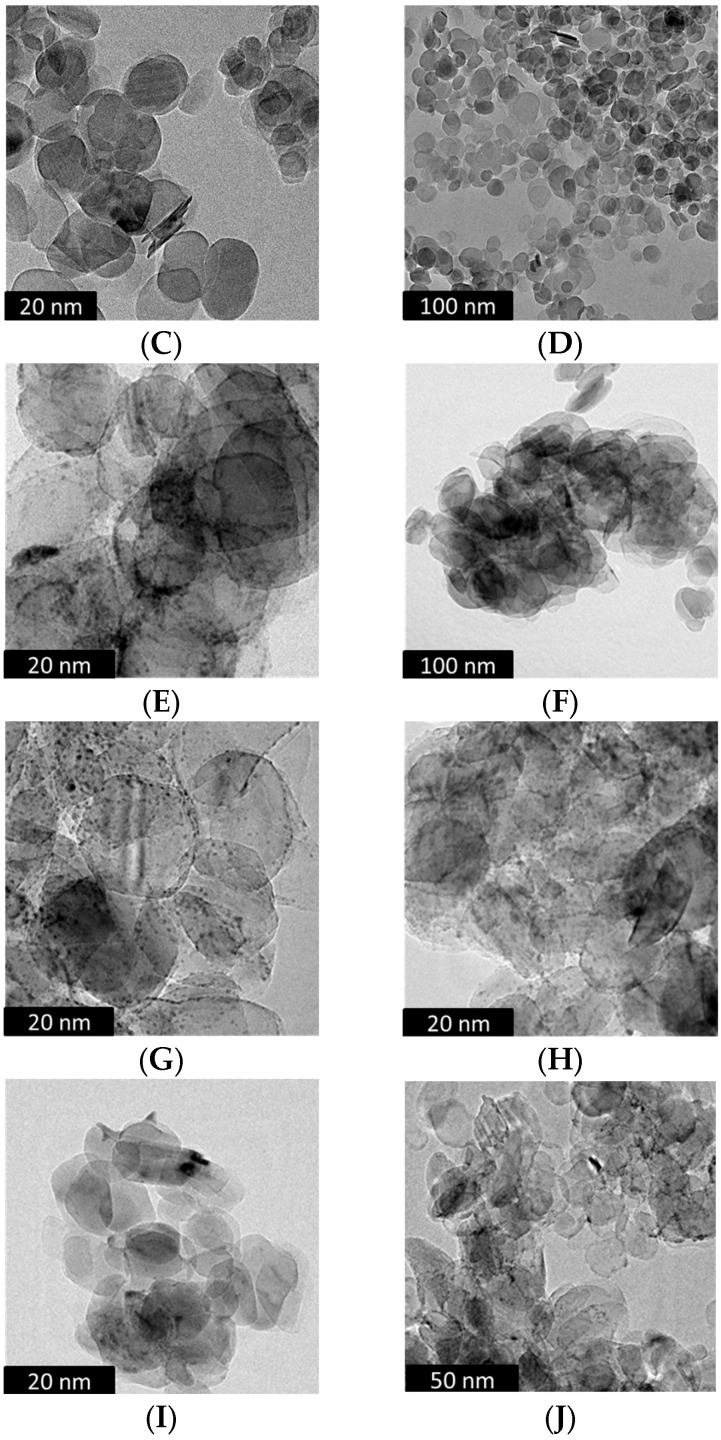
(**A**) TEM images of PbO nanoparticles and (**B**) the calculated size distribution of PbO nanoparticles from the TEM images. (**C**–**J**) TEM images of BN nanoparticles (**C**,**D**), NCPSPbBN17 (**E**,**F**), NCPSPbBN21 (**G**,**H**), and NCPSPbBN25 (**I**,**J**).

The polymer chains were linked together. The surface of the polymer is granular (Figure 6A) and porous (Figure S9) (magnified images from 1000 to 10,000) [46]. PS blocks linked with the macrocrosslinker PEG are continuous, forming the polymer phase and a branched structure. A structure was observed in which PS and PEG formed a homogeneous continuous matrix. The surface of NCPSPb3 has granular, rough, porous, voids and clusters (in Figure 6B and Figure S10). The size of the PbO nanoparticles on the NCPSPb3 surface is 352.11–456 nm. The morphological images of the PS-PEG/BN/PbO PCM nanocomposites are shown in Figures S3 and S9–S18. Structural (SEM, TEM) studies were conducted to examine the morphology, distribution of the nanoparticles, and changes in chemical structures of the PCM nanocomposites after thermal conductivity measurements. As a result of the analysis, it was observed that there were no structural aggregates in the nanocomposites and that the nanoparticles were homogeneously dispersed. The morphologies of PS-PEG/PbO, PS-PEG/BN, and the PS-PEG/BN/PbO PCM nanocomposites are presented using SEM images. In Figure 7, Figure 8, Figure 9 and Figure 10, the PS-PEG matrix showed smooth particles, cracks, voids, and porous surfaces, which took place in the presence of BN and PbO nanoparticles. In contrast, PS-PEG/BN/PbO PCM nanocomposites showed a rough and crumpled fracture structure, which was the result of local polymer deformation due to cracking from the addition of the BN nanoparticles and PbO nanoparticles. In Figure 7 and Figure 10, the BN nanoparticles and PbO nanoparticles were well dispersed in the PS-PEG matrix, and the compatibility between fillers and matrix was observed to be fine. When the content of BN nanoparticles and PbO nanoparticles was increased, the fillers formed a well-interconnected network in the PS-PEG matrix. In addition, at higher magnifications, the presence of cavities at the interactive interface of the BN/PbO nanoparticles and the polymer matrix was confirmed. In addition, these properties are effective factors that improve heat transfer in thermally conductive polymer composites.

SEM images of the NCPSPb8 nanocomposite are presented in Figure 7A and Figure S11 (magnified images from 1000 to 14,000). As the molecular weight of the crosslinker PEG in the PS-PEG (1500) copolymer increased, the roughness on the NCPSPb8 surface decreased, and a flatter surface was formed. When SEM photographs of 70 wt% PbO-doped nanocomposites are compared, the effect of the macrocrosslinker PEG molecular weight increase is seen. The size of the PbO particle on the NCPSPb8 surface is 233–720.7 nm (Figure 7A and Figure S11A–C). SEM images of NCPSPb13 have determined that pores (Figure 7B and Figure S12, magnified images from 1000 to 5000), granulation, branching, and agglomeration (in Figure 7B and Figure S12) are observed from the surface films of the composite, where the percentage of PbO is higher than the percentage of polymer. The size of the PbO particles on the surface was 9.266 nm in Figure 7B and 2.288–8.840 nm in Figure S12C. EDS images of the NCPSPb8 nanocomposite is presented in Figure 7C. EDS mapping and analysis of element distribution on the surface verified the presence of carbon (C) and lead (Pb) elements throughout the surface of the PS-PEG-PbO nanocomposite. The EDS spectra further showed the corresponding peaks of C and Pb in NCPSPb8 (Figure 7C).

SEM images of NCPSPbBN17 are shown in Figure 8A (magnified images from 5000 to 12,000). As seen in Figure 8A and Figure S13, the surfaces of the NCPSPbBN17 nanocomposite exhibit a morphological structure containing granular particles and metal compound particles (Figure 8A and Figure S13), pores, branching (Figure 8A and Figure S13A,B), aggregates, and cavities (in Figure 8A and Figure S13B,C). Nano-sized BN and PbO particles appear on the surface of the NCPSPbBN17 PCM nanocomposite. The magnitude of the BN and PbO particles is 235–409 nm. As seen from the SEM images shown in Figure 8B and Figure S14 (magnified images from 3000 to 10,000), the NCPSPbBN21 surface also has pores, particles, layers, branches, and voids, similar to the surface of the NCPS6 copolymer. BN and PbO nanoparticles appear on the surface of NCPSPbBN21; these have a particle size of 260–1400 nm (Figure 8B). EDS analysis of the NCPSPbBN21 composite shows Pb, B, C, O, and N elements on the surface of the composite (Figure 8C).

SEM images of the NCPSPbBN25 nanocomposite are shown in Figure 9A,B and Figure S15.

As seen in Figure 9A,B and Figure S15 (magnified images from 2500 to 15,000), when the amount of PbO added to PS-PEG (10,000) copolymer is increased to 70 wt%, PbO particles on the surface appear more intense. The particles, pores, and clusters on the surface of the composite can be seen from the SEM photographs in Figure 9A,B and Figure S15. The sizes of the BN and PbO nanoparticles on NCPSPBBN25 are 361–778 nm.

#### 3.4.2. TEM Results

Figure 10A,B shows the TEM images of PbO and BN nanoparticles. The PbO nanoparticles were in the range of 2–15 nm and the size distribution was very narrow. The TEM images showed the regular spherical shape and confirmed both their size and the homogenous size distribution. In addition, BN nanoparticles with a mean diameter as small as 100 nm could be obtained, as shown by TEM analysis (Figure 10C,D). The TEM photographs of the NCPSPbBN PCM nanomaterial are shown in Figure 10E–J. The small dark spots in the TEM images indicate the presence of BN and PbO nanoparticles that were bound to the copolymers. The TEM images of NCPSPbBN17 in Figure 10E,F are similar to the TEM images of NCPSPbBN21 and NCPSPbBN25, with BN nanoparticles and PbO nanoparticles dispersed in the interlocking spherical and rod-shaped copolymer structures, which appear as bright objects that are light in color.

#### 3.4.3. XRD Patterns of the BN Nanoparticle, PbO Nanoparticle, and the PS-PEG/BN/PbO PCM Nanocomposites

X-ray studies were taken to examine the nanoparticles and changes in chemical structures of the PCM nanocomposites after thermal conductivity measurements. It was observed that there were no chemical changes.

**Figure 11:**
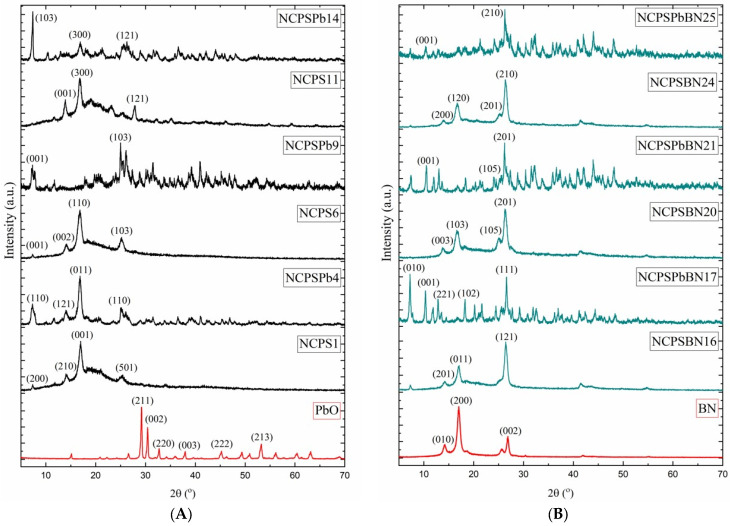
(**A**) XRD patterns of PbO, NCPS1, NCPSPb4, NCPS6, NCPSPb9, NCPS11, and NCPSPb14 PCM nanocomposites. (**B**) XRD patterns of BN, NCPSBN16, NCPSPbBN17, NCPSBN20, NCPSPbBN21, NCPSBN24, and NCPSPbBN25 PCM nanocomposites.

Figure 11 shows the XRD patterns of PbO, NCPS1, NCPSPb4, NCPS6, NCPSPb9, NCPS11, and NCPSPb14. The PbO nanoparticles were further analyzed by powder XRD. The diffractogram shown in Figure 11A is consistent with the nanostructure of PbO. The two strong peaks with 2θ values of 29.20° and 30.42° correspond to the (211) and (002) planes, respectively. The distance values between the planes corresponding to these values were determined as 3.056 and 2.936 Å, respectively. The other peaks of the nanostructure of PbO are 32.73°, 37.97°, 45.24°, and 53.24°, corresponding to Miller indices of (220), (003), (222), and (213), respectively. The distance values between the planes corresponding to 2θ = 32.73°, 37.97°, 45.24°, and 53.24° values are 2.733 Å, 2.367 Å, 2.002 Å, and 1.719 Å, respectively. The XRD patterns obtained for PbO are compatible with the literature [88,89].

The XRD patterns coded NCPS1, NCPS6, and NCPS11 in Figure 11A belong to the PS-PEG (1000, 1500, 10,000) block copolymer. Figure 11 shows X-ray diffraction patterns of the PS-PEG block copolymer, revealing spectra of a broad amorphous peak that appeared at 2θ = 20–24°. The sharp diffraction peak of the PEG part of the PS-PEG (1000) copolymer appeared at 17.00° (d = 5.211 Å) and 25.28° (d = 3.520 Å), which indicates a polymer with crystallinity. Miller indices of the sharp diffraction peaks of the PEG corresponding to 17.00 Å and 25.28° are (001), (501), respectively. X-ray diffraction patterns corresponding to 14.04° (d-spacing = 6.309 Å) belong to the PS part of the PS-PEG block copolymer, and Miller indices are (210) [90]. PEG, a semicrystalline polymer, and the PS network, an amorphous crosslinked polymer, produce a semicrystalline mixture when the molecular weight of PEG is changed in our experiments. For the molecular weight used in this study (PEG-1000, -1500, and -10,000), the two-component system is expected to be composed of amorphous blended regions, with some crystalline regions made up entirely of PEG [91]. The XRD patterns of BN, NCPSBN16, NCPSPbBN17, NCPSBN20, NCPSPbBN21, NCPSBN24, and NCPSPbBN25 nanocomposites are presented in Figure 11B. XRD diffraction peaks of BN nanostructures of (010), (200), and (002) h-BN are observed at 2θ = 14.24°, 17.02°, 26.88°. XRD structural analysis of the BN nanoparticles shows the highest peak at 17.02°, corresponding to Miller indices of (200) (Figure 11B). We determined that the 2θ value of the 100% peak value of BN is 17.02°, the d-spacing distance corresponding to this value is 5.205 Å, and the hkl value is (200). In some of the other reflections at 2θ = 30.40°, 41.86°, and 55.18°, the d-spacing distances detected in the XRD pattern corresponding to these values are 2.938 Å, 2.156 Å, and 1.663 Å, respectively, and this result correlates with the literature [92]. Looking at the graph in Figure 11B, it is seen that BN nanoparticles have a more crystalline structure compared with the PS-PEG polymer, and the BN nanoparticle has peak values close to the PS-PEG polymer. The XRD diffraction peaks of the NCPSBN16 nanocomposite PCM containing 50 wt% BN and 50 wt% PS-PEG block copolymer are 2θ = 7.29°, 14.20°, 17.08°, 26.44°, 54.66°, 75.86°, and 82.02°.

The XRD diffraction peaks belonging to BN nanoparticles are at 26.44° (d-spacing = 3.314 Å, and (121) Miller indices) and 55.18°d = 1.663 Å (see Figure 11B). The XRD patterns at 14.20° (d-spacing = 6.231 Å) and 17.08° (d-spacing = 5.187 Å) belong to the PEG macrocrosslinker part of the NCPSBN16 nanocomposite PCM. NCPSPbBN17 contains 15 wt% BN, 15 wt% PS-PEG, and 70 wt% PbO. In the XRD image of this sample, it is seen that the NCPSPbBN17 has a (100) peak value of 7.24°, and the corresponding interplanetary distance value is 12.201 Å while the hkl value is (010). As a result, when the SEM, TEM, and XRD results of the nanocomposites are analyzed, the size analysis and XRD patterns of the nanoparticles in the PCM nanocomposites gave results consistent with the literature. At the same time, F_c_ values varying in the range of 0.032–1.985% calculated from the DSC results of the PEG macrocrosslinker correspond to 2θ = 17.00°and 25.28°peaks on XRD charts. The XRD results show that the composites retain the crystallization structure of the PS-PEG polymer and nanoparticles; furthermore, there are only physical rather than chemical reactions between the PSPEG polymer and PBO and BN nanoparticles.

## 4. Conclusions

In the study, PCM nanocomposites containing PbO nanoparticles, BN nanoparticles, and PS-PEG copolymers were prepared. The characteristic analysis of materials was performed using DSC, TGA, SEM, and XRD methods. By examining the DSC curves, it is seen that the phase-change temperatures and enthalpy values of each material change with the change in the percentage contribution of the PS-PEG, BN nanoparticles, and PbO nanoparticles. These differences can be explained by the fact that phase-change materials provide new heat conduction paths, thereby changing the phase transition rate of the samples. In this study, PCM nanocomposites with latent heats ranging from 34.6 J g^−1^–67.6 J g^−1^ can be used to reduce indoor temperature fluctuations, improve indoor thermal comfort, and save electrical energy. At the same time, they can be used as building materials for precise temperature control and rapid temperature regulation conditions. The roles of particle/polymer and particle/particle interfaces on the thermal conductivity of PS-PEG DM/BN and PbO nanocomposites are discussed in detail, as well as the relationship between the thermal conductivity and the micro- and nano-structure of the composites. Recently, studies to improve the thermal conductivity of polymers have been directed toward the selective addition of nanofillers with high thermal conductivity properties. The thermal conductivity of the PS-PEG polymers is increased by PbO nanoparticle doping. It can be seen from the results that the thermal conductivity increases with increasing values of 0–60.90–70–90% of the PbO ratio. Higher thermal conductivity values were obtained for nanocomposites by adding nanoparticles such as BN and PbO to the PS-PEG polymer. Thermal conductivity values obtained from our materials show that they can be widely used in various engineering applications based on energy storage/release. PS-PG/PbO and PS-PG/PbO/BN PCM nanocomposites have emerged as versatile functional materials. PCM nanocomposites can be used for electrical and magnetic materials, EMI and Gamma radiation shielding, and reflecting and absorbing materials. The X-ray diffraction pattern corresponding to 2θ = 14.04° belongs to the PS part of the PS-PEG block copolymer and the Miller indices are (210). Two strong peaks of PbO nanoparticles were 2θ = 29.20° and 2θ = 30.42°, and the 2θ value of the 100% peak value of BN is 17.02°.

## Figures and Tables

**Table 1:** The content of the PCM nanocomposites.

PCM ID	PS-PEG	PS-PEG (wt%)	BN (wt%)	PbO (wt%)	Volume (mm^3^)
NCPS1	1000	100	0	0	575.3
NCPSPb2	1000	50	0	50	579.1
NCPSPb3	1000	30	0	70	523.9
NCPSPb4	1000	10	0	90	357.5
NCPSPb5	1000	46.2	0	53.8	753.8
NCPS6	1500	100	0	0	631.8
NCPSPb7	1500	50	0	50	734.5
NCPSPb8	1500	30	0	70	631.8
NCPSPb9	1500	10	0	90	463.6
NCPSPb10	1500	46.2	0	53.8	858.3
NCPS11	10,000	100	0	0	782.3
NCPSPb12	10,000	50	0	50	399.4
NCPSPb13	10,000	30	0	70	519.8
NCPSPb14	10,000	10	0	90	511.2
NCPSPb15	10,000	46.2	0	53.8	657.6
NCPSBN16	1000	50	50	0	696.5
NCPSPbBN17	1000	15	15	70	599.6
NCPSPbBN18	1000	5	5	90	551.1
NCPSPbBN19	1000	26.1	13	60.9	513.5
NCPSBN20	1500	50	50	0	591.3
NCPSPbBN21	1500	15	15	70	515.9
NCPSPbBN22	1500	5	5	90	638.3
NCPSPbBN23	1500	26.1	13	60.9	607.6
NCPSBN24	10,000	50	50	0	741.6
NCPSPbBN25	10,000	15	15	70	413.5
NCPSPbBN26	10,000	5	5	90	528.8
NCPSPbBN27	10,000	26.1	13	60.9	842.4

**Table 2:** The degradation temperatures and the remaining mass (wt%) of the PCM nanocomposites.

PCM ID	First Stage of Degradation		Second Stage of Degradation		Third Stage of Degradation		Fourth Stage of Degradation	
	t (°C)	Remaining Mass, %wt	t (°C)	Remaining Mass, %wt	t (°C)	Remaining Mass, %wt	t (°C)	Remaining Mass, %wt
NCPS1	46.7	76.7	254.2	62.5	343.5	49.4	381.2	36.4
NCPSPb2	40.3	99.2	249.6	88.4	335.4	71.4	382.8	45.1
NCPSPb3	49.0	99.9	244.4	83.1	289.0	71.8	375.3	42.3
NCPSPb4	43.3	98.7	85.9	88.73	241.8	83.7	402.9	51.8
NCPSPb5	80.8	71.5	257.9	66.3	383.7	21.2	-	-
NCPS6	53.4	93.5	322.4	82.5	417.9	0.5	-	-
NCPSPb7	50.7	92.7	257.6	64.8	280.7	58.4	386.9	22.7
NCPSPb8	39.9	97.5	243.3	83.4	374.0	38.0	428.7	33.4
NCPSPb9	47.2	98.1	165.9	85.9	349.0	61.0	370.1	53.9
NCPSPb10	47.2	90.51	85.7	81.9	253.4	76.7	380.0	30.4
NCPS11	31.2	96.1	66.5	76.0	293.8	69.3	401. 8	3.0
NCPSPb12	25.3	94.3	75.2	49.7	259.9	40.4	403.3	−54.0
NCPSPb13	41.6	97.3	239.1	89.5	326.4	71.2	371.9	48.8
NCPSPb14	44.0	97.6	231.9	87.2	287.7	77.2	372.0	56.2
NCPSPb15	45.1	98.4	245.1	91.4	372.2	64.6	424.9	61.1
NCPSBN16	46.5	98.8	254.8	96.4	366.9	63.4	424.8	60.0
NCPSPbBN17	54.7	97.04	233.7	93.2	331.7	77.1	381.5	69.2
NCPSPbBN18	37.1	97.5	228.3	91.6	370.0	65.2	420.1	62.7
NCPSPbBN19	47.2	87.9	230.8	84.6	331.1	12.6	393.5	3.9
NCPSBN20	37.0	99.0	281.9	95.3	376.6	63.4	418.7	61.2
NCPSPbBN21	39.2	97.8	237.4	88.7	344.0	68.5	371.9	61.5
NCPSPbBN22	53.8	99.0	152.9	97.0	357.9	72.0	421.0	69.4
NCPSPbBN23	41.2	97.1	242.8	86.9	365.1	58.6	427.0	53.2
NCPSBN24	51.8	99.2	272.8	96.6	385.3	75.9	417.3	74.8
NCPSPbBN25	64.7	96.5	235.5	92.6	361.9	68.7	410.8	66.7
NCPSPbBN26	65.8	99.0	236.7	95.1	377.2	68.8	412.4	68.0
NCPSPbBN27	28.3	99.2	238.5	95.1	371.7	54.7	421.8	50.8

**Table 3:** Thermal Properties of the PS-PEG PCM, PS-PEG/PbO PCM, and PS-PEG/BN/PbO PCM nanocomposites.

PCM ID	T_m_ (°C)	ΔH_m_ (J g^−1^)	T_c_ (°C)	ΔH_c_ (J g^−1^)	F_c_
NCPS1	73.2 185.2	3.7 7.31	7.9 76.8	1.24 1.31	0.032 0.034
NCPSPb3	81.8 185.8 200.7	67.6 1.68 4.62	188.4	5.12	0.728
NCPS6	80.6 133.1 159.5 185.4	4.16 0.69 0.31 0.40	78	2.27	0.063
NCPSPb8	59.9 83.0 203.0	8.01 43.8 26.7	185.5	4.94	0.724
NCPS11	58.8 137.2 190.1	12.6 13.3 4.96	31.5	7.98	0.305
NCPSPb13	62.1 83.2 123.2 204.7	7.38 27.2 6.64 21.4	186.2	5.18	0.698
NCPSBN16	73.8 137.3 186.1	2.75 0.58 4.69	4.7 89.4	1.67 1.14	0.089 0.087
NCPSPbBN17	57.8 83.8 186.8 204.7	7.27 34.6 0.96 10.4	190.2	5.36	1.985
NCPSBN20	80.3 185.3	1.81 1.15	11.5 93.2	1.04 0.67	0.094 0.092
NCPSPbBN21	59.4 84.4 123.2 204.7	18.8 22.2 31.5 27.6	187.4	5.44	1.777
NCPSBN24	55.5 184.8	6.73 8.49	112.1	0.62	0.053
NCPSPbBN25	60.0 84.1 106.1 125.4 205.8	11.9 10.01 6.07 6.05 21.6	188.6	3.04	0.860
PEO-CMC [79]	58.4–62.5	52.8–140.2	35.3–41.3	5.2–138	-
PEO-CEL [79]	62.5–63.4	40.6–134.7	32.5–39.5	40.2–127.3	-
AMPD/TAM [80]	114.7–122.6	5.1–181.5	19.3–187.1	20–203.8	-
NPG/TAM/PE/AMPD [80]	171.6	14.1	169.6	17.4	-
NPG/PE [81]	160.3–169.8	18.8–26.2	-	-	-
PE-TAM [82]	184.6	14.2	188.6	14.3	

## Data Availability

The data presented in this study are available on request from the corresponding author.

## Supplementary Materials

The following supporting information can be downloaded at: https://www.mdpi.com/article/10.3390/polym15102326/s1. Supplementary S1: TGA Measurements of the PS-PEG-PbO and -BN Nanocomposite PCMs; Supplementary S2: DSC Results of the PS-PEG/BN/PbO PCM Nanocomposites; Supplementary S3: The morphological images of the PS-PEG/BN/PbO PCM Nanocomposites.

## References

[1] Duraković B., Mešetović S. (2019). Thermal performances of glazed energy storage systems with various storage materials: An experimental study. Sustain. Cities Soc..

[2] Duraković B., Torlak M. (2017). Experimental and numerical study of a PCM window model as a thermal energy storage unit. Int. J. Low-Carbon Technol..

[3] Durakovic B., Torlak M. (2017). Simulation and experimental validation of phase change material and water used as heat storage medium in window applications. J. Mater. Environ. Sci..

[4] Farhat N., Inal Z. (2019). Solar thermal energy storage solutions for building application: State of the art. Herit. Sustain. Dev..

[5] Çakır A., Kurmuş E.F. (2019). Energy storage technologies for building applications. Herit. Sustain. Dev..

[6] Ermiş K., Findik F. (2020). Thermal energy storage. Sustain. Eng. Innov..

[7] Duraković B. (2020). PCM-Based Building Envelope Systems Innovative Energy Solutions for Passive Design.

[8] Teggar M., Arıcı M., Mezaache E.H., Mert M.S., Ajarostaghi S.S.M., Niyas H., Tuncbilek E., Ismail KA R., Younsi Z., Benhouia A. (2022). A comprehensive review of micro/nano-enhanced phase change materials. J. Therm. Anal. Calorim..

[9] Williams J.D., Peterson G. (2021). A review of thermal property enhancements of low temperature nano-enhanced phase change materials. Nanomaterials.

[10] Najim F.T., Mohammed H.I., Taqi Al-Najjar H.M., Thangavelu L., Mahmoud M.Z., Mahdi J.M., Tiji M.E., Yaïci W., Talebizadehsardari P. (2022). Improved melting of latent heat storage using fin arrays with non-uniform dimensions and distinct patterns. Nanomaterials.

[11] Chen K., Mohammed H.I., Mahdi J.M., Rahbari A., Cairns A., Talebizadehsardari P. (2022). Effects of non-uniform fin arrangement and size on the thermal response of a vertical latent heat triple-tube heat exchanger. J. Energy Storage.

[12] Hosseinzadeh K., Erfani Moghaddam M.A., Asadi A., Mogharrebi A.R., Jafari B., Hasani M.R., Ganji D.D. (2021). Effect of two different fins (longitudinal-tree like) and hybrid nano-particles (MoS_2_-TiO_2_) on solidification process in triplex latent heat thermal energy storage system. Alex. Eng. J..

[13] Talebizadehsardari P., Mohammed H.I., Mahdi J.M., Gillott M., Walker G.S., Grant D., Giddings D. (2021). Effect of airflow channel arrangement on the discharge of a composite metal foam-phase change material heat exchanger. Int. J. Energy Res..

[14] Ali H.M., Janjua M.M., Sajjad U., Yan W.M. (2019). A critical review on heat transfer augmentation of phase change materials embedded with porous materials/foams. Int. J. Heat Mass Transf..

[15] Moghaddam M.E., Abandani M.H.S., Hosseinzadeh K., Shafii M.B., Ganji D. (2022). Metal foam and fin implementation into a triple concentric tube heat exchanger over melting evolution. Theor. Appl. Mech. Lett..

[16] Bondareva N.S., Buonomo B., Manca O., Sheremet M.A. (2019). Heat transfer performance of the finned nano-enhanced phase change material system under the inclination influence. Int. J. Heat Mass Transf..

[17] Hosseinzadeh K., Montazer E., Shafii M.B., Ganji A. (2021). Solidification enhancement in triplex thermal energy storage system via triplets fins configuration and hybrid nanoparticles. J. Energy Storage.

[18] Hosseinzadeh K., Moghaddam M.E., Asadi A., Mogharrebi A., Ganji D. (2020). Effect of internal fins along with hybrid nano-particles on solid process in star shape triplex latent heat thermal energy storage system by numerical simulation. Renew. Energy.

[19] Ho C., Siao C.-R., Yang T.-F., Chen B.-L., Rashidi S., Yan W.-M. (2021). An investigation on the thermal energy storage in an enclosure packed with micro-encapsulated phase change material. Case Stud. Therm. Eng..

[20] Khedher N.B., Ghalambaz M., Alghawli A.S., Hajjar A., Sheremet M., Mehryan SA M. (2022). Study of tree-shaped optimized fins in a heat sink filled by solid-solid nanocomposite phase change material. Int. Commun. Heat Mass Transf..

[21] Khedher N.B., Bantan R.A., Kolsi L., Omri M. (2022). Performance investigation of a vertically configured LHTES via the combination of nano-enhanced PCM and fins: Experimental and numerical approaches. Int. Commun. Heat Mass Transf..

[22] Meng L., Ivanov A.S., Kim S., Zhao X., Kumar N., Young-Gonzales A., Saito T., Bras W., Gluesenkamp K., Bocharova V. (2022). Alginate−Sodium Sulfate Decahydrate Phase Change Composite with Extended Stability. ACS Appl. Polym. Mater..

[23] Peng G., Dou G., Hu Y., Sun Y., Chen Z. (2020). Review Article Phase Change Material (PCM) Microcapsules for Thermal Energy Storage. Adv. Polym. Technol..

[24] Mohaddes F., Islam S., Shanks R., Fergusson M., Wang L., Padhye R. (2014). Modification and evaluation of thermal properties of melamine-formaldehyde/n-eicosane microcapsules for thermo-regulation applications. Appl. Therm. Eng..

[25] Yuzhan L., Navin K., Jason H., Damilola O.A., Kai L., Turnaoglu T., Monojoy G., Rios O., Tim JLaClair Samuel G., Kyle R.G. (2022). Stable salt hydrate-based thermal energy storage materials. Compos. Part B.

[26] Han W., Ge C., Zhang R., Ma Z., Wang L., Zhang X. (2019). Boron nitride foam as a polymer alternative in packaging phase change materials: Synthesis, thermal properties and shape stability. Appl. Energy.

[27] Sundararajan S., Samui A.B., Kulkarni P.S. (2018). Synthesis and characterization of poly(ethylene glycol) acrylate (PEGA) copolymers for application as polymeric phase change materials (PCMs). React. Funct. Polym..

[28] Anuar Sharif M.K., Al-Abidi A.A., Mat S., Sopian K., Ruslan M.H., Sulaiman M.Y., Rosli M.A.M. (2015). Review of the application of phase change material for heating and domestic hot water systems. Renew. Sustain. Energy Rev..

[29] Deng Y., Li J., Qian T., Guan W., Li Y., Yin X. (2016). Thermal conductivity enhancement of polyethylene glycol/expanded vermiculite shape-stabilized composite phase change materials with silver nanowire for thermal energy storage. Chem. Eng. J..

[30] Wang W., Yang X., Fang Y., Ding J., Yan J. (2009). Enhanced thermal conductivity and thermal performance of form-stable composite phase change materials by using β-Aluminum nitride. Appl. Energy.

[31] Song S., Qiu F., Zhu W., Guo Y., Zhang Y., Ju Y., Feng R., Liu Y., Chen Z., Zhou J. (2019). Polyethylene glycol/halloysite@Ag nanocomposite PCM for thermal energy storage: Simultaneously high latent heat and enhanced thermal conductivity. Sol. Energy Mater. Sol. Cells.

[32] Chen J., Huang X., Sun B., Jiang P. (2019). Highly Thermally Conductive Yet Electrically Insulating Polymer/Boron Nitride Nanosheets Nanocomposite Films for Improved Thermal Management Capability. ACS Nano.

[33] Jiang Y., Shi X., Feng Y., Li S., Zhou X., Xie X. (2018). Enhanced thermal conductivity and ideal dielectric properties of epoxy composites containing polymer modified hexagonal boron nitride. Compos. Part A Appl. Sci. Manuf..

[34] Wu M.Q., Wu S., Cai Y.F., Wang R.Z., Li T.X. (2021). Form-stable phase change composites: Preparation, performance, and applications for thermal energy conversion storage and management. Energy Storage Mater..

[35] Meng Q., Hu J. (2008). A poly(ethylene glycol)-based smart phase change material. Sol. Energy Mater. Sol. Cells.

[36] Liu B., Zhang X., Ji J. (2021). Review on solar collector systems integrated with phase-change material thermal storage technology and their residential applications. Int. J. Energy Res..

[37] Dash L., Mahanwar P.A. (2021). A Review on Organic Phase Change Materials and Their Applications. Int. J. Eng. Appl. Sci. Technol..

[38] Chen C., Wang L., Huang Y. (2009). Crosslinking of the electrospun polyethylene glycol/cellulose acetate composite fibers as shape-stabilized phase change materials. Mater. Lett..

[39] Chen C., Wang L., Huang Y. (2009). Ultrafine electrospun fibers based on stearyl stearate/polyethylene terephthalate composite as form stable phase change materials. Chem. Eng. J..

[40] Chen C., Wang L., Huang Y. (2008). A novel shape-stabilized PCM: Electrospun ultrafine fibers based on lauric acid/polyethylene terephthalate composite. Mater. Lett..

[41] Al-Migdady A.K., Jawarneh A.M., Ababneh A.K., Dalgamoni H.N. (2021). Numerical Investigation of the Cooling Performance of PCM-based Heat Sinks Integrated with Metal Foam Insertion. Jordan J. Mech. Ind. Eng..

[42] Nomura T., Okinaka N., Akiyama T. (2010). Waste heat transportation system, using phase change material (PCM) from steelworks to chemical plant. Resour. Conserv. Recycl..

[43] Gracia D.A., Rincón L., Castell A., Jiménez M., Boer D., Medrano M., Cabeza L.F. (2010). Life cycle assessment of the inclusion of phase change materials (PCM) in experimental buildings. Energy Build..

[44] Tyagi V.V., Buddhi D. (2007). PCM thermal storage in buildings: A state of art. Renew. Sustain. Energy Rev..

[45] Jin J., Lin F., Liu R., Xiao T., Zheng J., Qian G., Liu H., Wen P. (2017). Preparation and thermal properties of mineral-supported polyethylene glycol as form-stable composite phase change materials (CPCMs) used in asphalt pavements. Sci. Rep..

[46] Tang B., Qiu M., Zhang S. (2012). Thermal conductivity enhancement of PEG/SiO_2_ composite PCM by in situ Cu doping. Sol. Energy Mater. Sol. Cells.

[47] Meng X., Zhang H., Sun L., Xu F., Jiao Q., Zhao Z., Zhang J., Zhou H., Sawada Y., Liu Y. (2013). Preparation and thermal properties of fatty acids/CNTs composite as shape-stabilized phase change materials. J. Therm. Anal. Calorim..

[48] Feng L., Song P., Yan S., Wang H., Wang J. (2015). The shape-stabilized phase change materials composed of polyethylene glycol and graphitic carbon nitride matrices. Thermochim. Acta.

[49] Sun Q., Yuan Y., Zhang H., Cao X., Sun L. (2017). Thermal properties of polyethylene glycol/carbon microsphere composite as a novel phase change material. J. Therm. Anal. Calorim..

[50] Pielichowska K., Pielichowski K. (2014). Phase change materials for thermal energy storage. Prog. Mater. Sci..

[51] Yuan Y., Zhang H., Zhang N., Sun Q., Cao X. (2016). Effect of water content on the phase transition temperature, latent heat and water uptake of PEG polymers acting as endothermal-hydroscopic materials. J. Therm. Anal. Calorim..

[52] Wensel J., Wright B., Thomas D., Douglas W., Mannhalter B., Cross W., Hong H., Kellar J. (2008). Enhanced thermal conductivity by aggregation in heat transfer nanofluids containing metal oxide nanoparticles and carbon nanotubes. Appl. Phys. Lett..

[53] Alizadeh N., Broughton R.M., Auad M.L. (2021). Graft Semi-Interpenetrating Polymer Network Phase Change Materials for Thermal Energy Storage. ACS Appl. Polym. Mater..

[54] Sharma R.K., Ganesan P., Tyagi V.V., Mahlia T.M.I. (2016). Accelerated thermal cycle and chemical stability testing of polyethylene glycol (PEG) 6000 for solar thermal energy storage. Sol. Energy Mater. Sol. Cells.

[55] Zhang L., Zhu J., Zhou W., Wang J., Wang Y. (2012). Thermal and electrical conductivity enhancement of graphite nanoplatelets on form-stable polyethylene glycol/polymethyl methacrylate composite phase change materials. Energy.

[56] Mo S., Mo B., Wu F., Jia L., Chen Y. (2021). Preparation and thermal performance of ternary carbonates/silica microcomposites as phase change materials. J. Sol-Gel Sci. Technol..

[57] Zhang C., Shi Z., Li A., Zhang Y.F. (2020). RGO-Coated Polyurethane Foam/Segmented Polyurethane Composites as Solid–Solid Phase Change Thermal Interface Material. Polymers.

[58] Chen B., Han M., Zhang B., Ouyang G., Shafei B., Wang X., Hu S. (2019). Efficient Solar-to-Thermal Energy Conversion and Storage with High-Thermal-Conductivity and Form-Stabilized Phase Change Composite Based on Wood-Derived Scaffolds. Energies.

[59] Cui Y., Liu C., Hu S., Yu X. (2011). The experimental exploration of carbon nanofiber and carbon nanotube additives on thermal behavior of phase change materials. Sol. Energy Mater. Sol. Cells.

[60] Wu B., Chen R., Fu R., Agathopoulos S., Su X., Liu H. (2020). Low thermal expansion coefficient and high thermal conductivity epoxy/Al2O3/T-ZnOw composites with dual-scale interpenetrating network structure. Compos. Part A Appl. Sci. Manuf..

[61] Qi G.Q., Liang C.L., Bao R.Y., Liu Z.Y., Yang W., Xie B.H., Yang M.B. (2014). Polyethylene glycol based shape-stabilized phase change material for thermal energy storage with ultra-low content of graphene oxide. Sol. Energy Mater. Sol. Cells.

[62] Shen J., Zhang P., Song L., Li J., Ji B., Li J., Chen L. (2019). Polyethylene glycol supported by phosphorylated polyvinyl alcohol/graphene aerogel as a high thermal stability phase change material. Compos. Part B Eng..

[63] Barani Z., Mohammadzadeh A., Geremew A., Huang C.Y.T., Coleman D., Mangolini L., Kargar F., Baladin A.A. (2019). Thermal Properties of the Binary-Filler Hybrid Composites with Graphene and Copper Nanoparticles. Adv. Funct. Mater..

[64] Tu J., Li H., Cai Z., Zhang J., Hu X., Huang J., Xiong C., Jiang M., Huang L. (2019). Phase change-induced tunable dielectric permittivity of poly(vinylidene fluoride)/polyethylene glycol/graphene oxide composites. Compos. Part B Eng..

[65] Hu H. (2020). Recent advances of polymeric phase change composites for flexible electronics and thermal energy storage system. Compos. Part B Eng..

[66] Yang J., Tang L.S., Bao R.Y., Bai L., Liu Z.Y., Yang W., Xie B.H., Yang M.B. (2017). Largely enhanced thermal conductivity of poly(ethylene glycol)/boron nitride composite phase change materials for solar-thermal-electric energy conversion and storage with very low content of graphene nanoplatelets. Chem. Eng. J..

[67] Guerra V., Wan C., McNally T. (2019). Thermal conductivity of 2D nano-structured boron nitride (BN) and its composites with polymers. Prog. Mater. Sci..

[68] Jia X., Li Q., Ao C., Hu R., Xia T., Xue Z., Wang Q., Deng X., Zhang W., Lu C. (2020). High thermal conductive shape-stabilized phase change materials of polyethylene glycol/boron nitride@chitosan composites for thermal energy storage. Compos. Part A Appl. Sci. Manuf..

[69] Lu X., Huang H., Zhang X., Lin P., Huang J., Sheng X., Zhang L., Qu J. (2019). Novel light-driven and electro-driven polyethylene glycol/two-dimensional MXene form-stable phase change material with enhanced thermal conductivity and electrical conductivity for thermal energy storage. Compos. Part B Eng..

[70] Kenisarin M., Mahkamov K. (2007). Solar energy storage using phase change materials. Renew. Sustain. Energy Rev..

[71] Huang C., Qian X., Yang R. (2018). Thermal conductivity of polymers and polymer nanocomposites. Mater. Sci. Eng. R Rep..

[72] Zhou W., Qi S., An Q., Zhao H., Liu N. (2007). Thermal conductivity of boron nitride reinforced polyethylene composites. Mater. Res. Bull..

[73] Presley M.A., Christensen P.R. (1997). Thermal conductivity measurements of particulate materials 1. A review. J. Geophys. Res. Planets.

[74] Cinan Z.M., Baskan T., Erol B., Mutlu S., Misirlioglu Y., Savaskan Yilmaz S., Yilmaz A.H. (2021). Gamma irradiation, thermal conductivity, and phase change tests of the cement-hyperbranched poly amino-ester-block-poly cabrolactone-polyurathane plaster-lead oxide and arsenic oxide composite for development of radiation shielding material. Int. J. Energy Res..

[75] Cinan Z.M., Erol B., Baskan T., Mutlu S., Savaskan Yilmaz S., Yilmaz A.H. (2021). Gamma Irradiation and the Radiation Shielding Characteristics: For the Lead Oxide Doped the Crosslinked Polystyrene-b-Polyethyleneglycol Block Copolymers and the Polystyrene-b-Poly ethylene glycol-Boron Nitride Nanocomposites. Polymers.

[76] Savaskan Yilmaz S. (1994). Synthesis and Investigation of Ion Exchange Properties of New Ion Exchangers. Ph.D. Thesis.

[77] Savaşkan S., Besirli N., Hazer B. (1996). Synthesis of some new cation-exchanger resins. J. Appl. Polym. Sci..

[78] Zeighampour F., Khoddami A., Hadadzadeh H., Ghane M. (2022). Thermal conductivity enhancement of shape-stabilized phase change nanocomposites via synergistic effects of electrospun carbon nanofiber and reduced graphite oxide nanoparticles. J. Energy Storage.

[79] Pielichowska K., Pielichowski K. (2011). Biodegradable PEO/cellulose-based solidsolid phase change materials. Polym. Adv. Technol..

[80] Gao W., Lin W., Liu T., Xia C. (2007). An experimental study on the heat storage performances of polyalcohols NPG, TAM, PE, and AMPD and their mixtures as solid-solid phase-change materials for solar energy applications. Int. J. Green Energy.

[81] Wang X., Lu E., Lin W., Liu T., Shi Z., Tang R., Wang C. (2000). Heat storage performance of the binary systems neopentyl glycol/pentaerythritol and neopentyl glycol/trihydroxy methylaminomethane as solid–solid phase change materials. Energy Convers. Manag..

[82] Fallahi A., Guldentops G., Tao M., Granados-Focil S., Van Dessel S. (2017). Review on solid-solid phase change materials for thermal energy storage: Molecular structure and thermal properties. Appl. Therm. Eng..

[83] Tritt T.M. (2004). Physics of Solids and Liquids, Thermal Conductivity Theory, Properties, and Applications.

[84] Pan W., Phillpot S.R., Wan C., Chernatynskiy A., Qu Z. (2012). Low thermal conductivity oxides. Camb. Univ. Press.

[85] Pierson H.O. (1993). Handbook of Carbon, Graphite, Diamond and Fullerences: Properties, Processing and Applications.

[86] Wypych G. (2000). Handbook of Fillers: Physical Properties of Fillers and Filled Materials.

[87] Fischer J.E. (2006). Carbon nanotubes: Structure and properties. Carbon Nanomaterials.

[88] Lebedev S.M. (2021). A comparative study on thermal conductivity and permittivity of composites based on linear low-density polyethylene and poly(lactic acid) filled with hexagonal boron nitride. Polym. Compos..

[89] Osman A.F., El Balaa H., El Samad O., Awad R., Badawi M.S. (2023). Assessment of X-ray shielding properties of polystyrene incorporated with different nano-sizes of PbO. Radiat. Environ. Biophys..

[90] Zeng S., Liang Y., Lu H., Wang L., Dinh X.Y., Yu X., Ho H.P., Hu X., Yong K.T. (2012). Synthesis of symmetrical hexagonal-shape PbO nanosheets using gold nanoparticles. Mater. Lett..

[91] Hussein A.M., Dannoun E.M.A., Aziz S.B., Brza M.A., Abdulwahid R.T., Hussen S.A., Rostam S., Mustafa D.M.T., Muhammad D.S. (2020). Steps Toward the Band Gap Identification in Polystyrene Based Solid Polymer Nanocomposites Integrated with Tin Titanate Nanoparticles. Polymers.

[92] Li Y., Ma Q., Huang C., Liu G. (2013). Crystallization of Poly (ethylene glycol) in Poly (methyl methacrylate) Networks. Express Polym. Lett..

