# Supplementary material for: Thermal Conductivity and Phase-Change Properties of Boron Nitride–Lead Oxide Nanoparticle-Doped Polymer Nanocomposites
Source: Polymers (Basel). 2023 May 16;15(10):2326. doi: 10.3390/polym15102326 (PMC10220655; doi:10.3390/polym15102326)
Supplement: Supplementary file 1 [file polymers-15-02326-s001.zip › polymers-2321804-supplementary.docx]

Supplementary Material: Thermal Conductivity and Phase Change Properties of Boron Nitride-Lead Oxide Nanoparticles Doped Polymer Nanocomposites

Bülend Ortaç, Saliha Mutlu, Taylan Baskan, Sevil Savaskan Yilmaz, Ahmet Hakan Yilmaz and Burcu Erol

S1.TGA Measurements of the PS-PEG-PbO and -BN Nanocomposite PCMs







**(A) (B)**







**(C) (D)**





**(E)**

**Figure S1.** TGA thermograms of the PCM nanocomposites: (A) NCPS1, (B) NCPSPb2, (C) NCPSPb3, (D) NCPSPb4, (E) NCPSPb5.







**(A) (B)**







**(C) (D)**





**(E)**

**Figure S2.** TGA thermograms of PCM nanocomposites : (A) NCPS6, (B) NCPSPb7, (C) NCPSPb8, (D) NCPSPb9, (E) NCPSPb10.







**(A) (B)**







**(C) (D)**





**(E)**

**Figure S3.** TGAs of researched PCMs: (A) NCPS11, (B) NCPSPb12, (C) NCPSPb13, (D) NCPSPb14, (E) NCPSPb15.







**(A) (B)**







**(C) (D)**

**Figure S4.** TGA thermograms of the PCM nanocomposites: (A) NCPSBN16, (B) NCPSPbBN17, (C) NCPSPbBN18, (D) NCPSPbBN19.







**(A) (B)**







**(C) (D)**

**Figure S5.** TGA thermograms of researched PCMs: (A) NCPSBN20, (B) NCPSPbBN21, (C) NCPSPbBN22, (D) NCPSPbBN23.







**(A) (B)**







**(C) (D)**

**Figure S6.** TGA thermal lines of researched PCMs: (A) NCPSBN24, (B) NCPSPbBN25, (C) NCPSPbBN26, (D) NCPSPbBN27.

S2.DSC Results of the PS-PEG/BN/PbO PCM Nanocomposites.


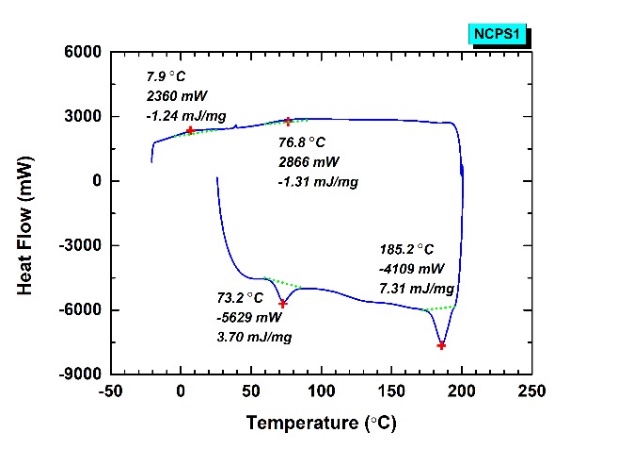

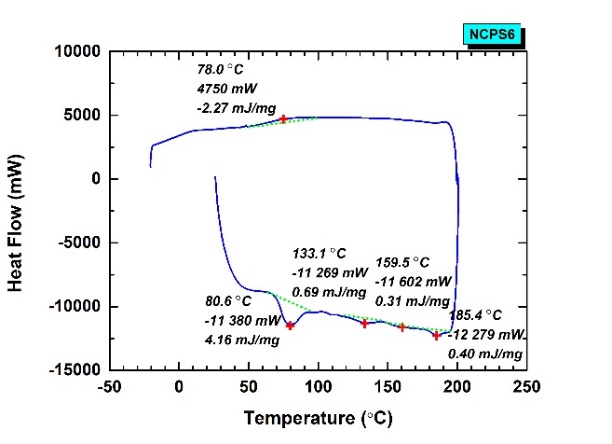


**(A) (B)**


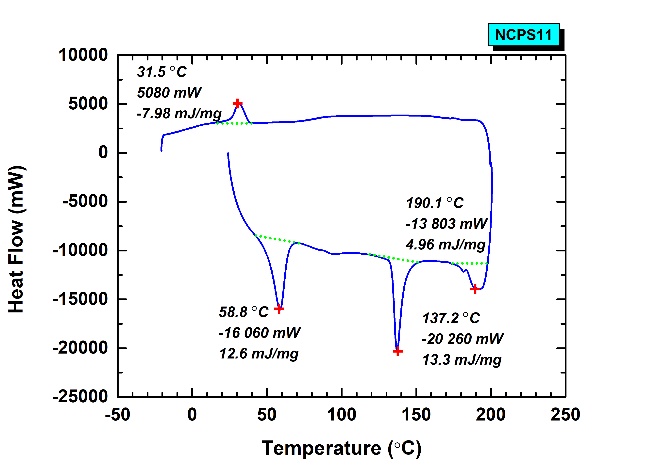


**(C)**

**Figure S7.** Thermal behavior of PS-PEG PCMs: (A) NCPS1, (B) NCPS6, (C) NCPS11.


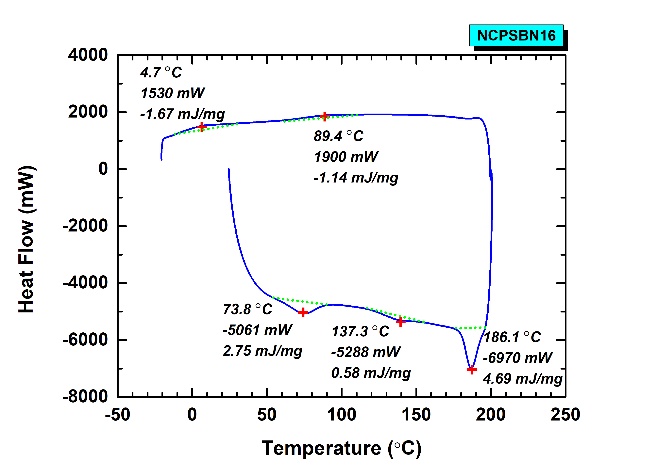

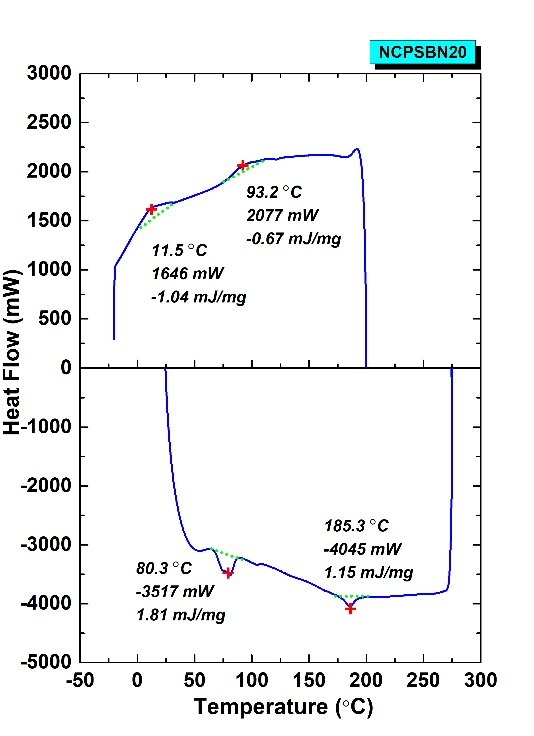


**(A) (B)**


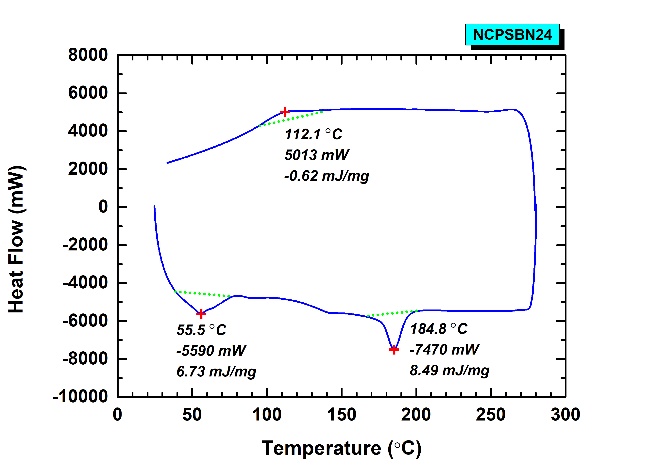


**(C)**

**Figure S8.** Thermal behavior of PS-PEG PCM Nanocomposites: (A) NCPSBN16, (B) NCPSBN20, (C) NCPSBN24.

S3. The morphological images of the PS-PEG/BN/PbO PCM Nanocomposites

**
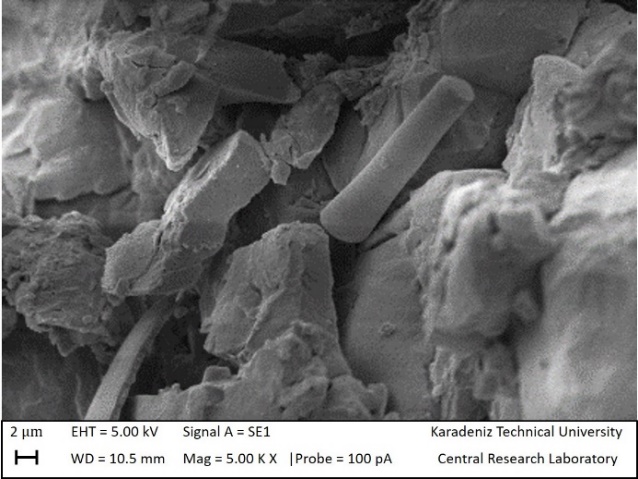

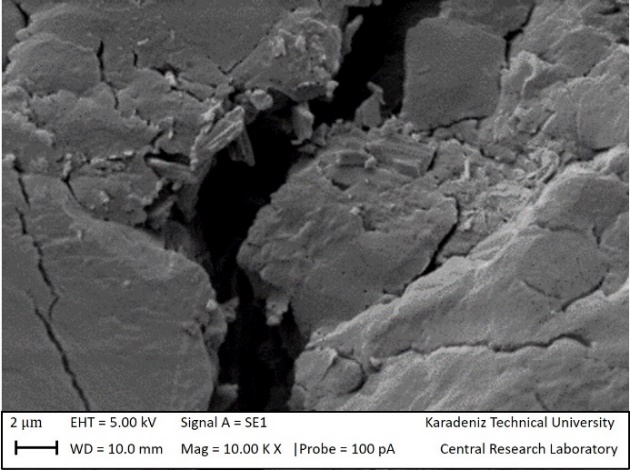
**

**(A) (B)**

**
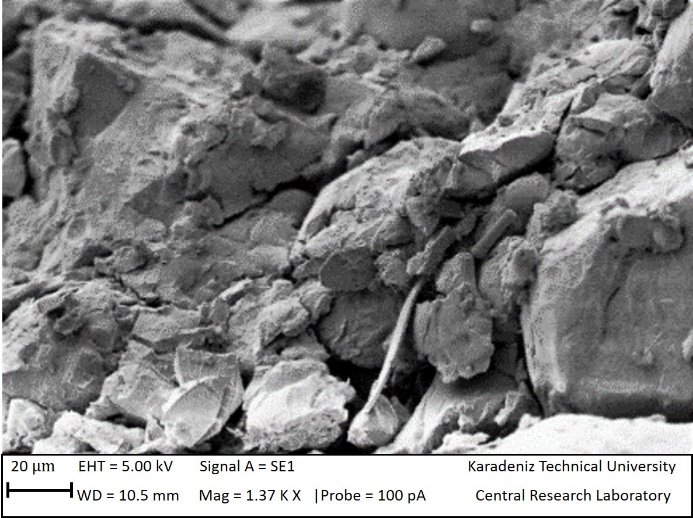
**

**(C)**

**Figure S9.** SEM images of the NCPS6.


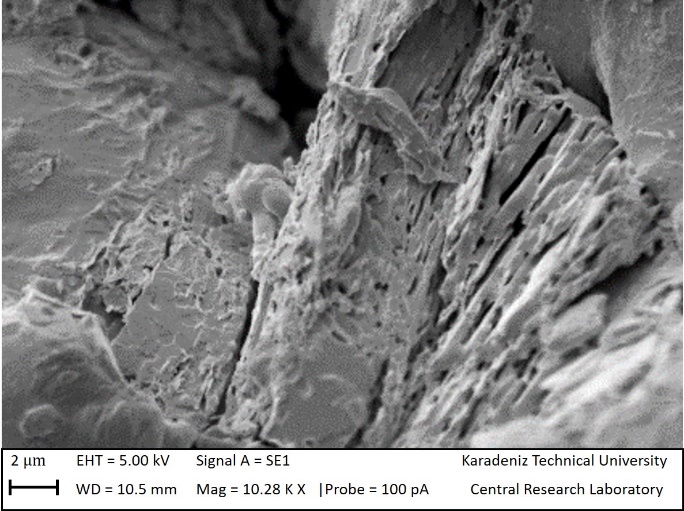

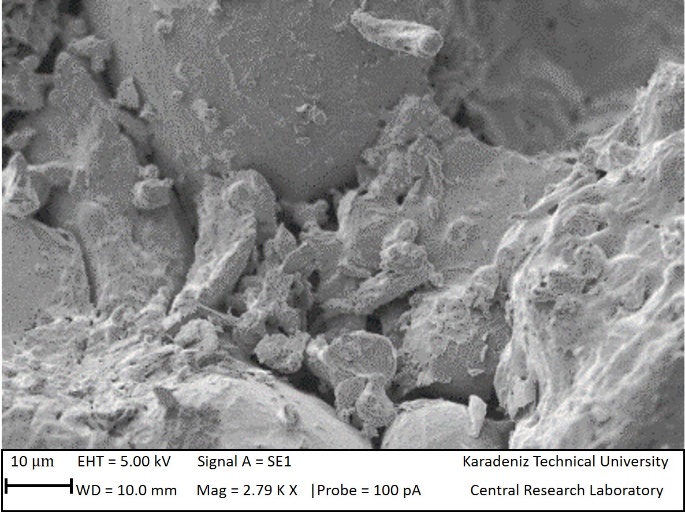


**(A) (B)**

**
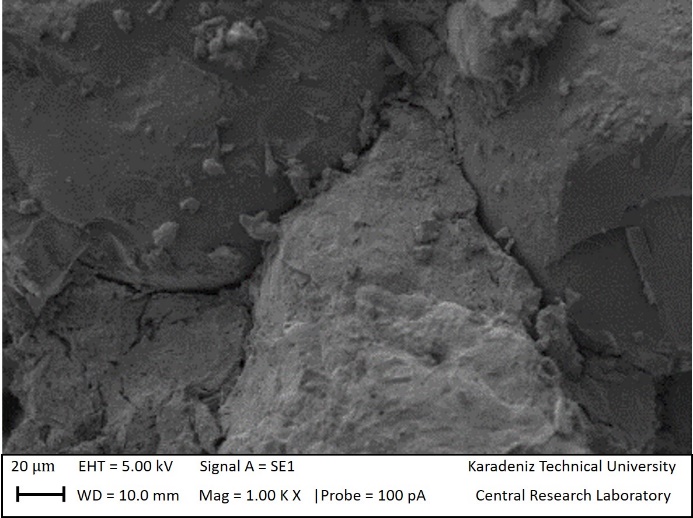
**

**(C)**

**Figure S10.** SEM images of the NCPSPb3 (magnified images from 2000 to 20000).


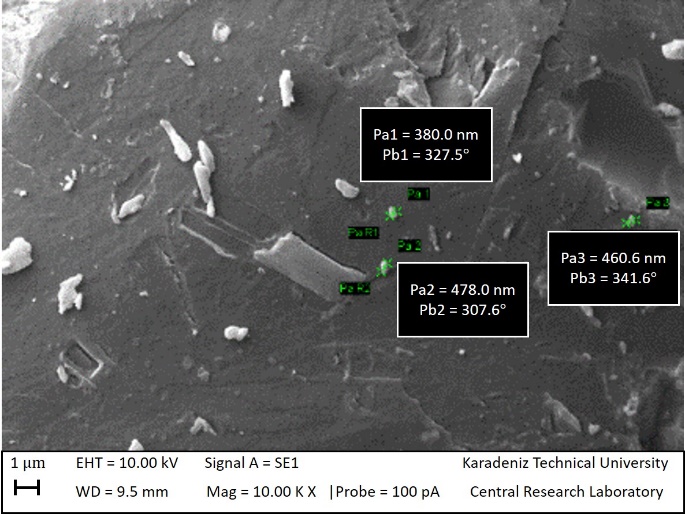

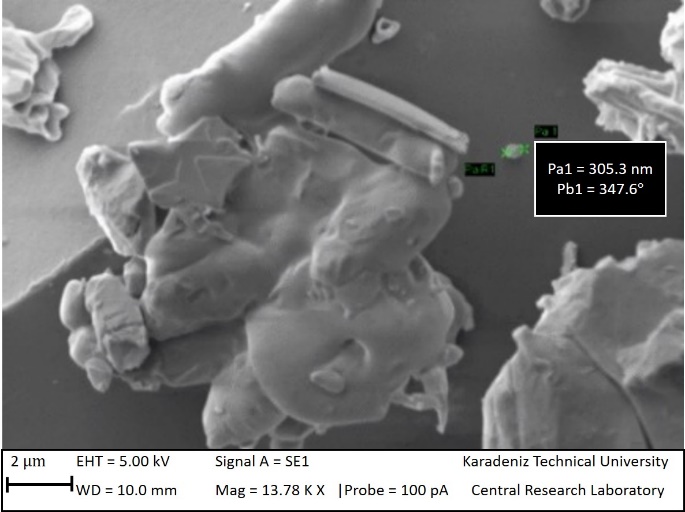


1. **(B)**

**
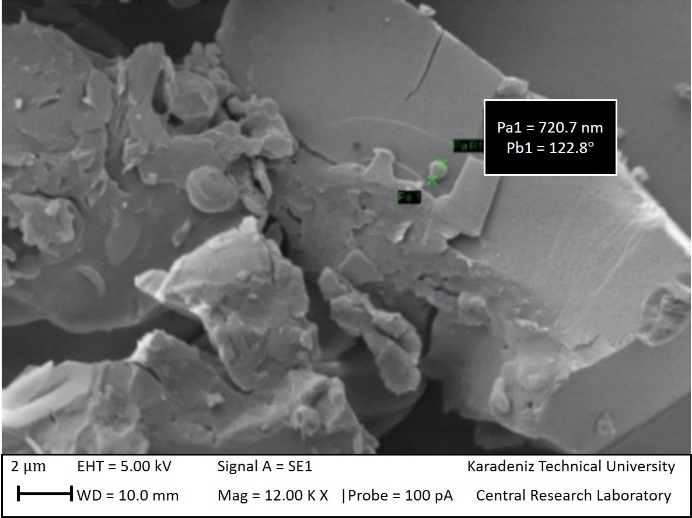
**

**(C)**


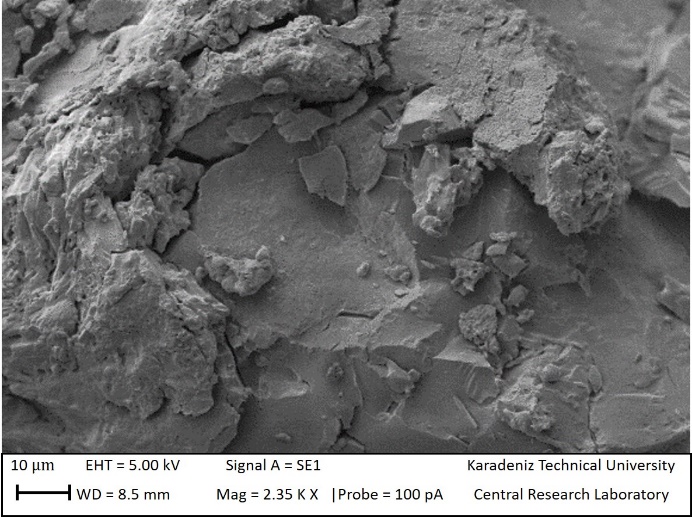

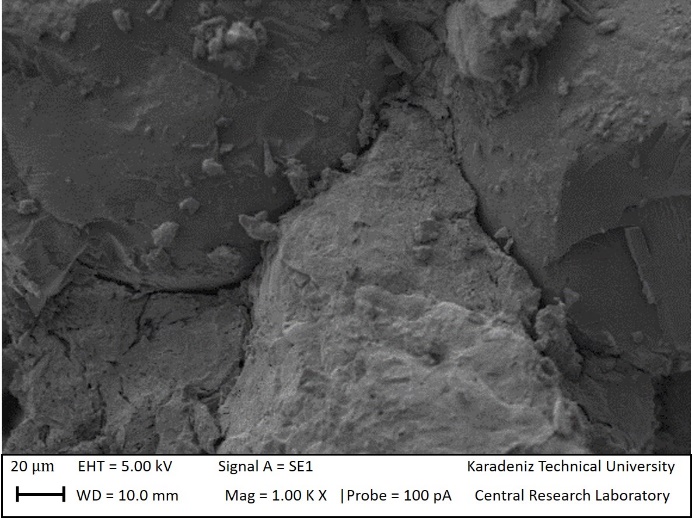


**(D) (E)**

**Figure S11.** SEM images of the NCPSPb8.


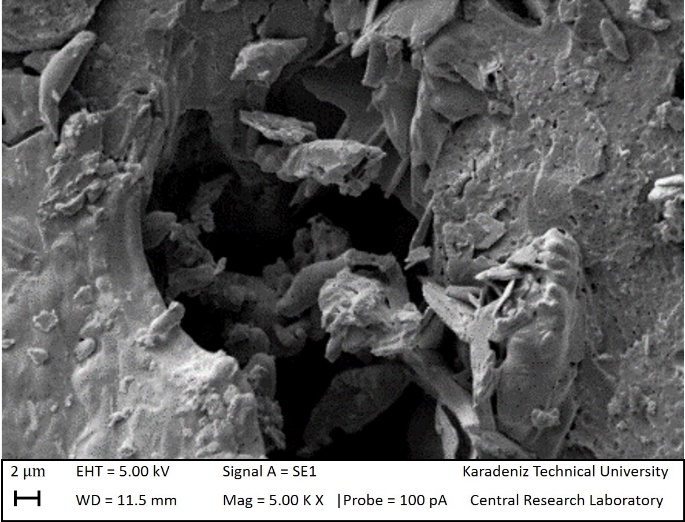

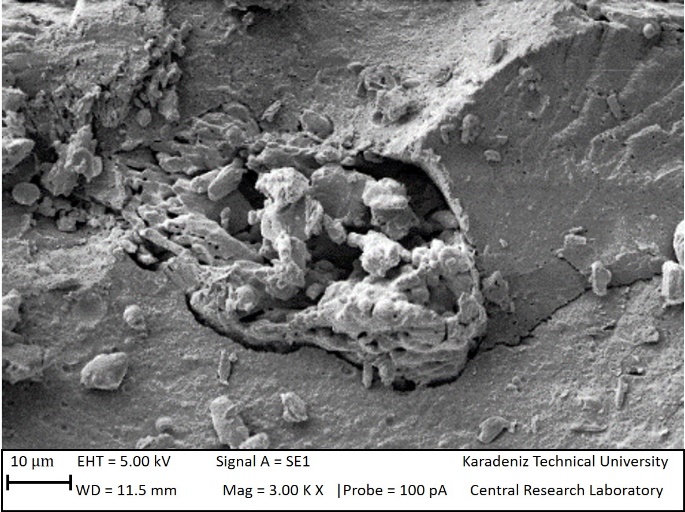


**(A) (B)**

**
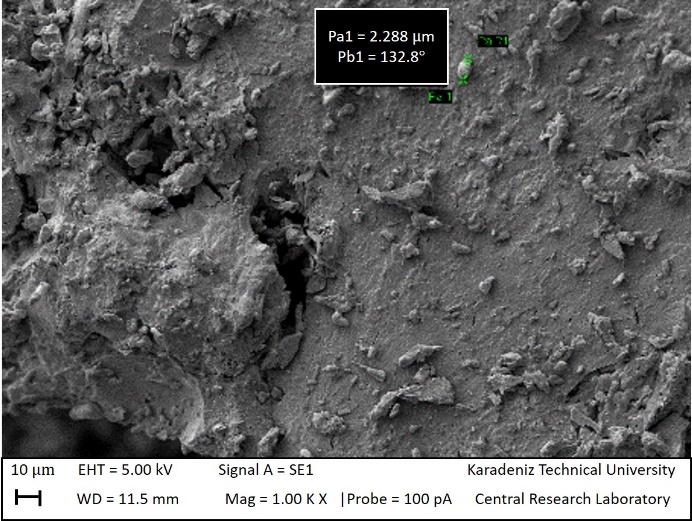
**

**(C)**

**Figure S12.** SEM images of the NCPSPb13 (magnified images from 1000 to 5000).


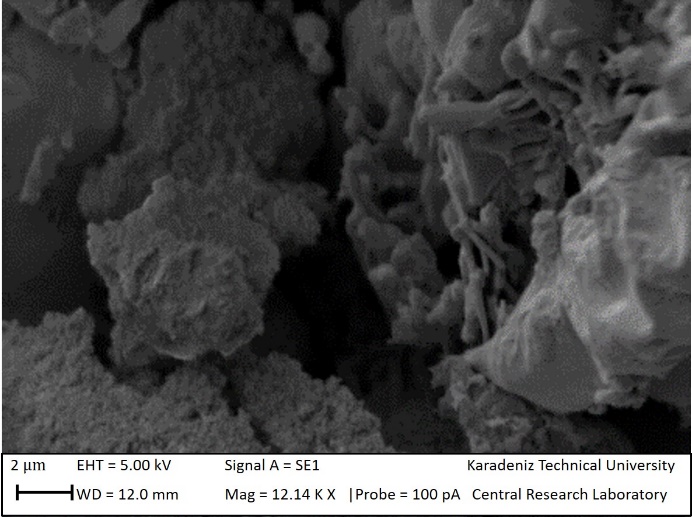

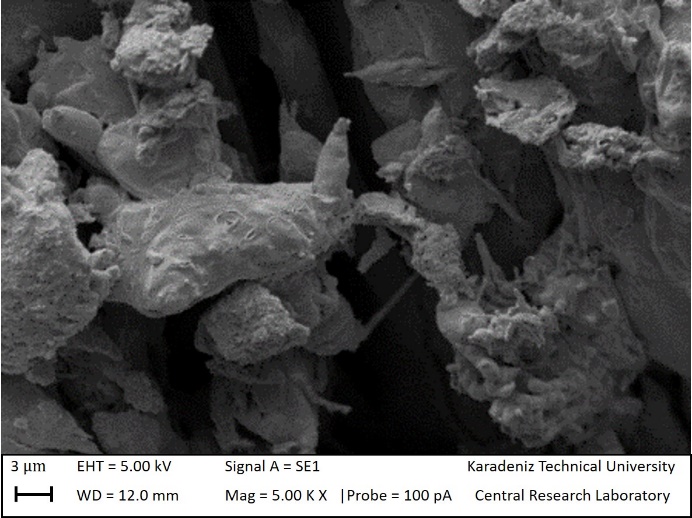


**(A) (B)**

**
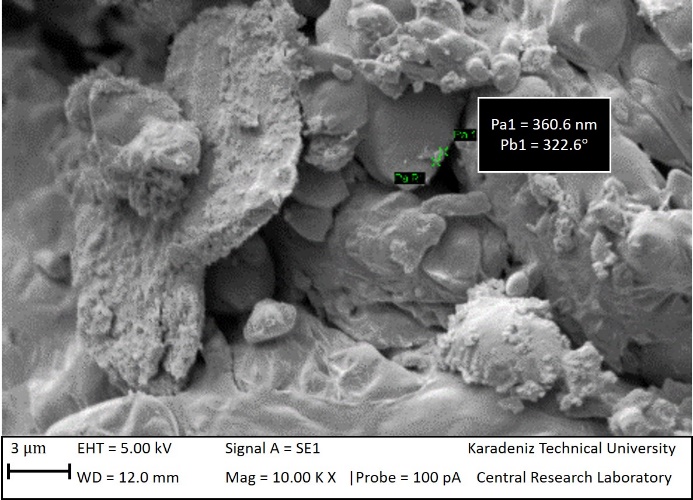
**

**(C)**

**Figure S13.** SEM images of the NCPSPbBN17.

**
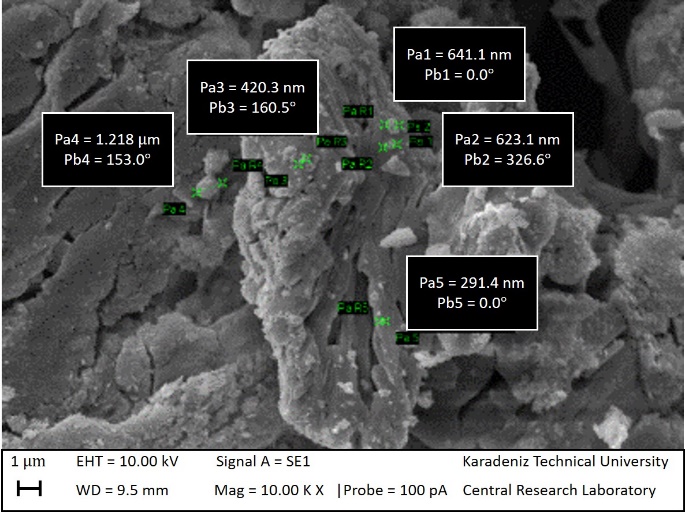

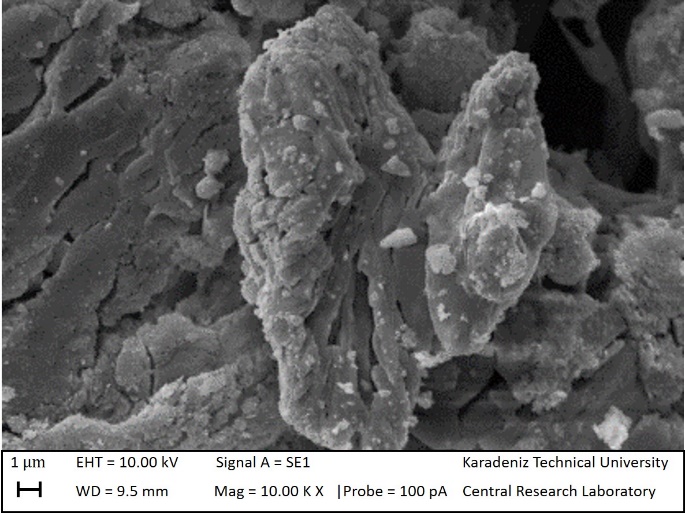
**

**(A) (B)**

**
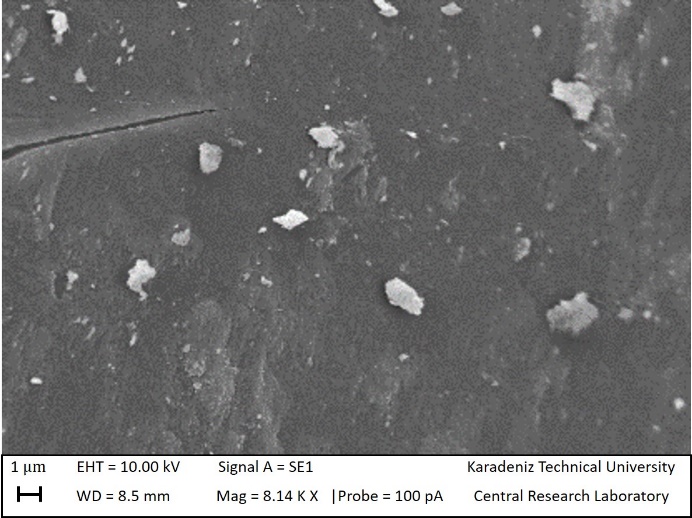
**

**(C)**

**
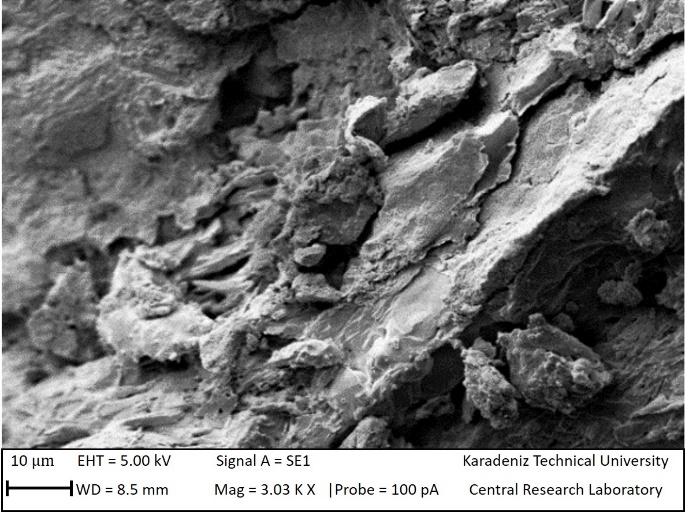

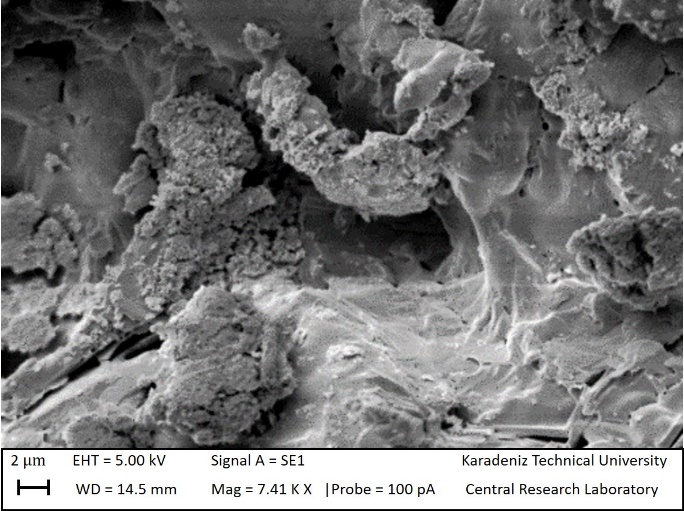
****(D) (E)**

**Figure S14.** SEM images of the NCPSPbBN21.


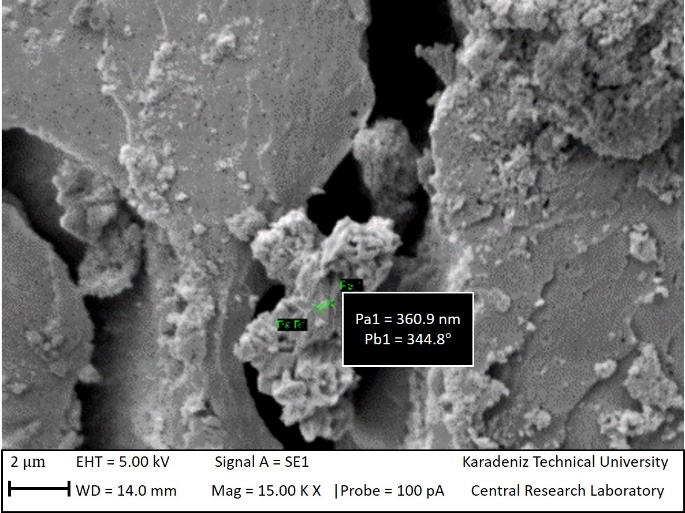

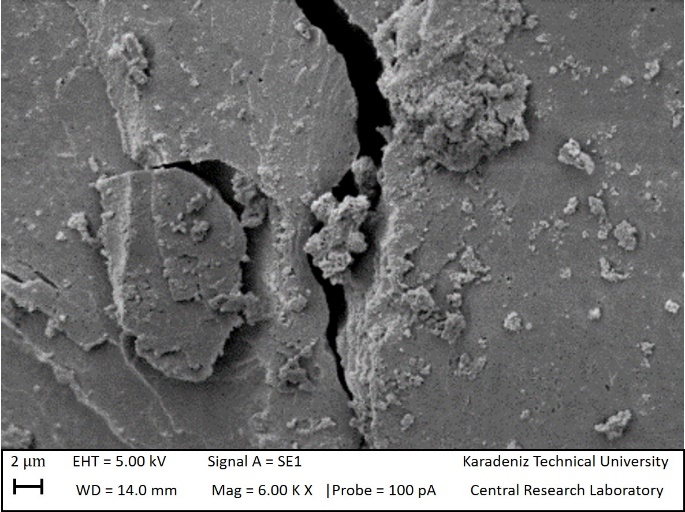


**(A) (B)**

**
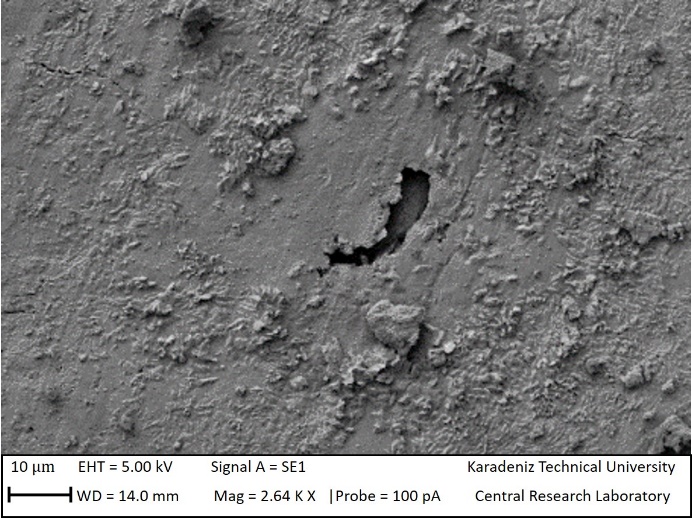
**

**(C)**

**Figure S15.** SEM images of the NCPSPbBN25.


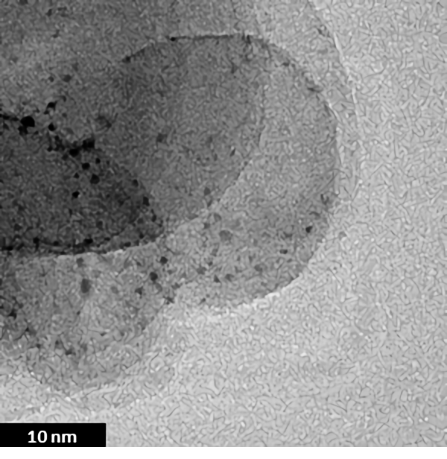

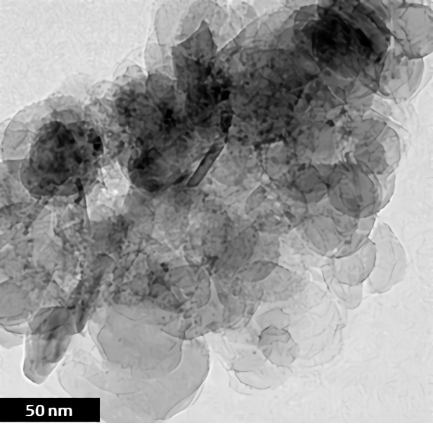

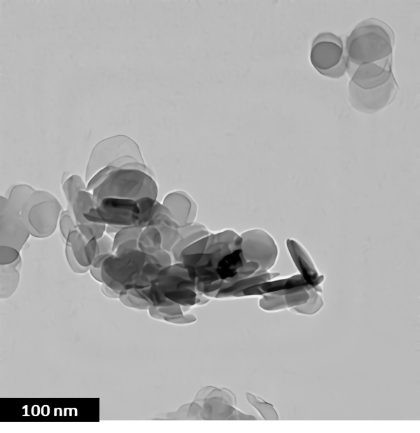


**(A) (B) (C)**

**Figure S16.** TEM images of NCPSPbBN25.

**
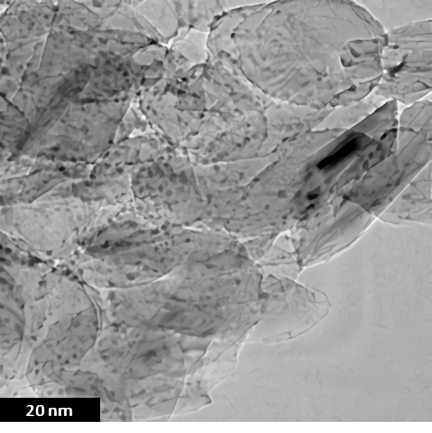
**
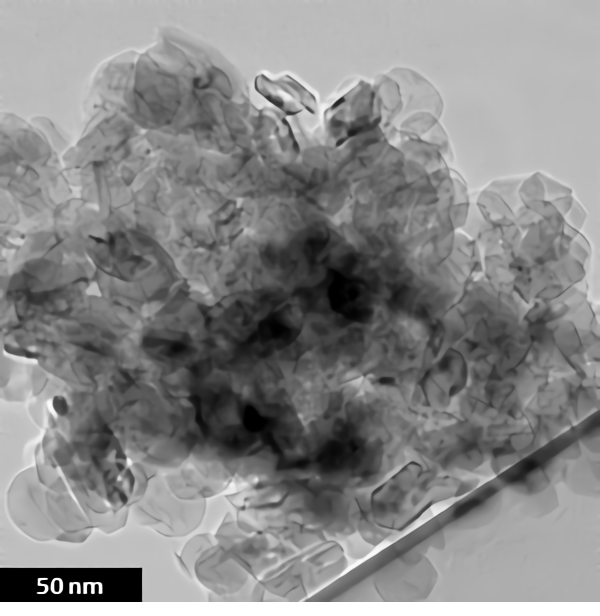

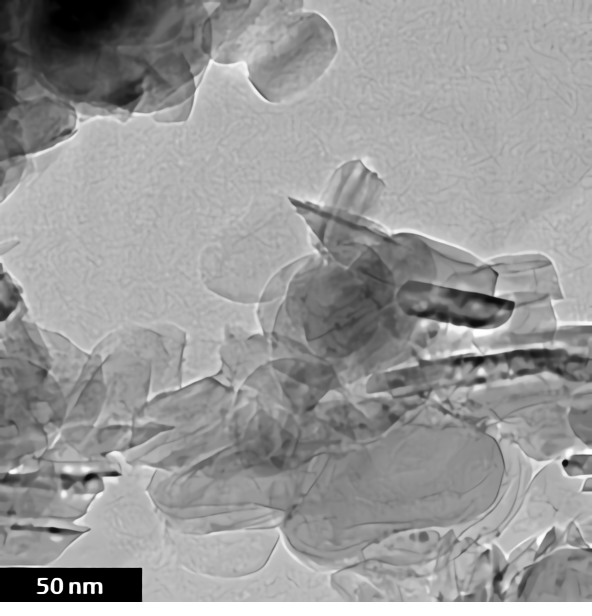


**(A) (B) (C)**

**Figure S17.** TEM images of NCPSPbBN21.


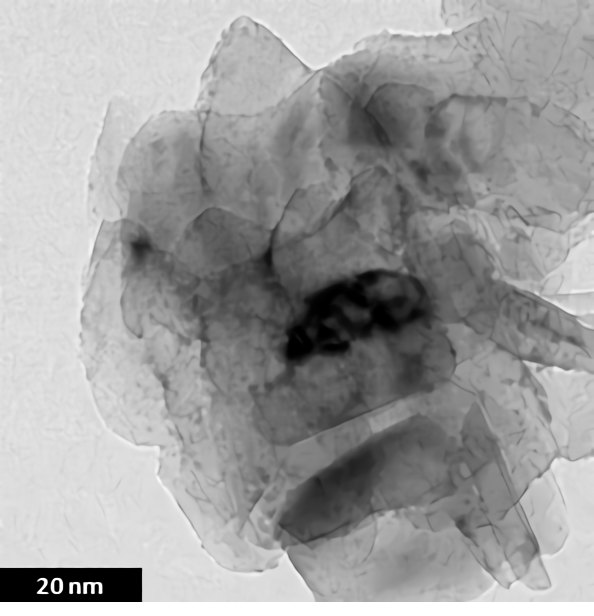

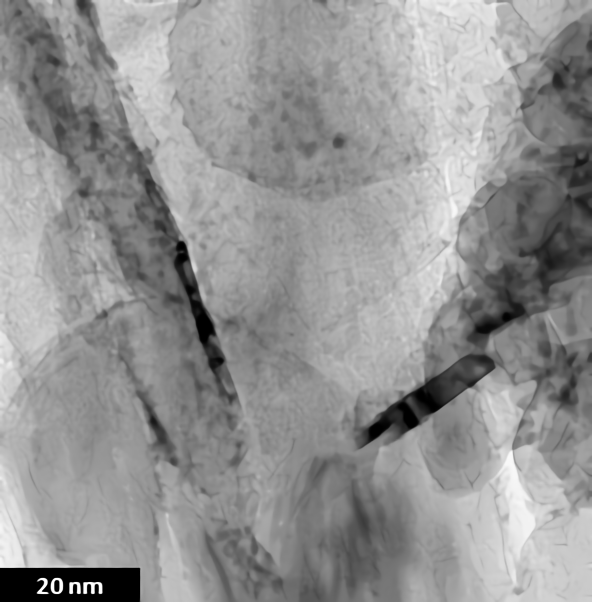

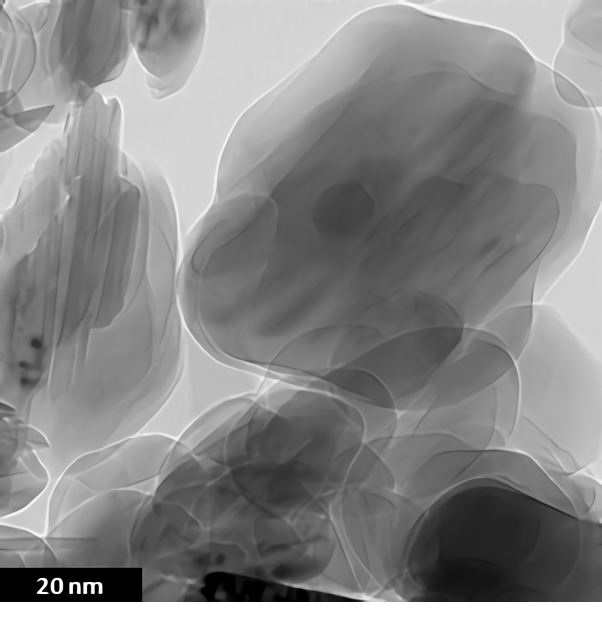


**(A) (B) (C)**

**Figure S18.** TEM images of NCPSPbBN17 .
